# Antidepressant-like Effects of Representative Types of Food and Their Possible Mechanisms

**DOI:** 10.3390/molecules28196992

**Published:** 2023-10-09

**Authors:** Jingjing Piao, Yingwei Wang, Tianqi Zhang, Jiayu Zhao, Qianyu Lv, Mengyu Ruan, Qin Yu, Bingjin Li

**Affiliations:** 1 Jilin Provincial Key Laboratory for Molecular and Chemical Genetics, The Second Hospital of Jilin University, Changchun 130041, China; piaojj22@mails.jlu.edu.cn (J.P.); zhangtq9920@mails.jlu.edu.cn (T.Z.); zhaojy7019@mails.jlu.edu.cn (J.Z.); lvqy7019@mails.jlu.edu.cn (Q.L.); ruanmy9920@mails.jlu.edu.cn (M.R.); yuqin9921@mails.jlu.edu.cn (Q.Y.); 2 Engineering Laboratory for Screening of Antidepressant Drugs, Jilin Province Development and Reform Commission, Changchun 130041, China; 3 Changchun Zhuoyi Biological Co., Ltd., Changchun 130616, China; 13384489608@163.com; 4 Jilin Provincial Key Laboratory on Target of Traditional Chinese Medicine with Anti-Depressive Effect, Changchun 130041, China

**Keywords:** depression, neurotransmitter, diet, antidepressant, brain-derived neurotrophic factor

## Abstract

Depression is a mental disorder characterized by low mood, lack of motivation, negative cognitive outlook, and sleep problems. Suicide may occur in severe cases, although suicidal thoughts are not seen in all cases. Globally, an estimated 350 million individuals grapple with depression, as reported by the World Health Organization. At present, drug and psychological treatments are the main treatments, but they produce insufficient responses in many patients and fail to work at all in many others. Consequently, treating depression has long been an important topic in society. Given the escalating prevalence of depression, a comprehensive strategy for managing its symptoms and impacts has garnered significant attention. In this context, nutritional psychiatry emerges as a promising avenue. Extensive research has underscored the potential benefits of a well-rounded diet rich in fruits, vegetables, fish, and meat in alleviating depressive symptoms. However, the intricate mechanisms linking dietary interventions to brain function alterations remain largely unexplored. This review delves into the intricate relationship between dietary patterns and depression, while exploring the plausible mechanisms underlying the impact of dietary interventions on depression management. As we endeavor to unveil the pathways through which nutrition influences mental well-being, a holistic perspective that encompasses multidisciplinary strategies gains prominence, potentially reshaping how we approach and address depression.

## 1. Introduction

Depression is a mental disorder primarily distinguished by low mood and reduced interest in normally reinforcing activities as the primary clinical characteristics. These symptoms are also characterized by changes in cognition that are typified by a negative global outlook that disengages the patient from daily life, as well as being associated with negative expectations for the future. Depression can harm patients to varying degrees, from loss of interest and anhedonia in mild cases of depression, to self-harm and suicide in more severe cases. Therefore, depression has been of wide concern for society and is treated with medication and psychotherapy [1,2]. While the majority of current antidepressants are based on the monoamine hypothesis, their effectiveness varies among individuals and often comes with specific side effects [3]. While medication treatment is the main intervention for depression in older people, their vulnerability to medication side effects is heightened [4]. Table 1 provides an overview of the possible mechanisms underpinning the actions of antidepressant medications. Acknowledging the limitations of drug therapy, attention has turned to alternative primary or secondary interventions, including dietary interventions, to protect vulnerable populations better [5,6]. Nutritional psychiatry is one of the most widely considered approaches. Studies have shown that foods can affect central nervous system (CNS) function, reduce the risk of depression, and improve depression by altering levels of neurotransmitters or their function, reducing inflammation, and regulating the hypothalamic–pituitary–adrenal (HPA) axis through gut-brain peptides, and perhaps through the regulation of gut microbiota [7,8,9].

## 2. The Pathogenesis of Depression

Depression is a common mental disease, but the etiological mechanisms are complex, multifaceted, and not completely understood. Several neurotransmitter systems, brain-derived neurotrophic factor (BDNF), glial cells, inflammation, and neuroendocrine systems have all been implicated in the development of depression. Reduced function of several neurotransmitters was initially associated with depression, primarily based on indirect evidence related to the mechanism of action of most antidepressant drugs. In 1965, Bunney proposed the monoamine neurotransmitter hypothesis, initially focusing on serotonin (5-hydroxytryptamine, 5-HT) and norepinephrine (NE), although some later versions of the hypothesis included dopamine (DA), which suggested that reduced monoamine function in the brain was closely related to the occurrence of depression [26]. Most early and current antidepressant drugs act to upregulate the function of one or more monoamine neurotransmitters, especially 5-HT [27,28]. The most recent study found that monoamine neurotransmitters regulate fundamental emotions and predominantly affect depressive disorder (MDD), indicating that monoamine neurotransmitters still play important roles in depression [29,30]. However, there has always been a delay in the antidepressant actions of these drugs, despite immediate effects on monoamine neurotransmission, suggesting that some indirect effect of increasing monoamine levels is the key mechanism of their antidepressant responses [31]. Depression and antidepressant-like effects also involve other neurotransmitters as part of the neurocircuitry underlying aspects of emotion, motivation, and cognition that are affected by depressive illness. Research has found that the incidence of depression is also related to the dysfunction of glutamate (Glu) and gamma-aminobutyrate (GABA) systems [32]. Many antidepressants may act initially by increasing the concentration of neurotransmitters in the synapses, but downstream mediators may have even greater importance [33,34,35]. Studies have shown that BDNF can promote neuronal survival after antidepressant treatment and plays a crucial role in the mechanisms of antidepressant actions [36,37]. In addition, several other mechanisms may interact with different stages of the depression process. Depression is considered a microglial disorder [38]. Abnormal activation of glial cells may contribute to depression-related manifestations [39]. In 1995, Maes proposed that depression is closely associated with inflammation [40]. In addition, the HPA axis is an essential part of the neuroendocrine system, and dysregulation of the HPA axis is a significant mediator of the development and expression of depression [41,42]. Recently, some research found that gut–brain peptides and the state of the gut microbiota are also involved in depression [43,44].

## 3. Role of Neurotransmitter Systems in Depression

### 3.1. Serotonin (5-hydroxytryptamine, 5-HT)

The synthesis of 5-HT is catalyzed by tryptophan hydroxylase (TPH), which has two subtypes, TPH1 and TPH2. TPH is the rate-limiting enzyme that catalyzes the formation of the essential amino acid tryptophan (Trp) into 5-hydroxytryptophan (5-HTP), and then 5-HT is formed by aromatic amino acid decarboxylase [45,46]. The 5-HT has a vital role in regulating mood [47]. The 5-HT system is a target for regulating mood disturbance caused by chronic social stress or other causes [48]. Low levels of 5-HT are associated with sleep disorders and circadian rhythm disturbances in depression [49,50]. The effects of 5-HT-are produced by activation of different 5-HT receptor subtypes, including 5-HT1A, 5-HT1B, and 5-HT2A, which have some role in depression and antidepressant responses [51]. The 5-HT1A receptor is a G protein-coupled receptor with inhibitory properties. Both pre and postsynaptic 5-HT1A receptors are involved in the pathogenesis of depression [52,53]. Animal and human studies have reported that loss of the hippocampal 5-HT1A binding site is associated with depression or depression-like behavior [54,55,56]. Current studies have found that both 5-HT1A agonists and 5-HT1A antagonists have similar effects to antidepressants [57,58]. The 5-HT1A agonists (YL-0919, brexpiprazole, and NLX-101) stimulate 5-HT1A postsynaptic heteroreceptors to increase 5-HT levels, whereas 5-HT1A antagonists (MIN-117) increase 5-HT levels by inhibiting 5-HT1A presynaptic autoreceptors [58]. Receptors for 5-HT1B are mainly distributed in the CNS. Female mice lacking 5-HT1B receptors had shorter immobility times in the forced swimming test (FST) and tail suspension test (TST) [59]. Different 5-HT1B agonists were observed to reduce immobility time in the FST in mouse depression models [60]. In a genetic animal model of depression, Flinders Sensitive Line (FSL) rats have significant reductions in 5-HT synthesis in the frontal cortex, hippocampus (HP), and thalamus [61]. When the 5-HT system is dysfunctional, and the activity and release of 5-HT are inhibited, the antidepressant effect can be exerted by regulating the synaptic concentration of 5-HT in the HP [62]. The 5-HT signaling at 5-HT2A receptors (5-HT2AR) is associated with many psychiatric and neurodegenerative disorders, with the prefrontal cortex acting as a central player and 5-HT2ARs highly expressed in the medial prefrontal cortex (mPFC), regulating cortical activity [63]. Trp is a precursor of 5-HT, and low doses of Trp supplements have been shown to improve depression rapidly [64]. A low Trp diet induces a decrease in 5-HT content in the CNS, and consequently, the impaired 5-HT function may contribute to developing diseases associated with depression [65].

### 3.2. Norepinephrine (NE)

NE is involved in emotional and cognitive functions and is a critical neurotransmitter in the CNS. Tyrosine hydroxylase (TH) is the rate-limiting enzyme in the NE synthesis pathway, and dopamine β-hydroxylase (DBH) is essential for NE biosynthesis [66,67]. Treatment with antidepressants such as desipramine, escitalopram, or duloxetine reversed NE levels and improved monoaminergic transmission in rats with chronic mild stress (CMS) depression [68]. Tricyclic antidepressants such as desipramine inhibit the reuptake of monoamine neurotransmitters by the presynaptic membrane of nerve endings and play an antidepressant role, although it is important to remember that although the effects on NE neurotransmission are immediate, clinical effects typically take several weeks to occur [69]. Nonetheless, evidence suggests that NE is likely to play an essential role in the development and progression of MDD and that delayed symptoms of depression are associated with the norepinephrine transporter (NET) gene [70,71]. The antidepressant-like effects of venlafaxine, a 5-HT and NE reuptake inhibitor (SNRI), may be associated with abnormal synthesis and normalization of metabolism of monoamine neurotransmitters in a mouse model of chronic unpredictable stress (CUS) [72]. The antidepressant SNRI also selectively increased NE levels in the hypothalamus [73]. The Glu modulator ketamine also produces antidepressant-like effects and also modulates peripheral and central brain neurotransmitter systems such as NE, which may be involved in its antidepressant-like effects [74]. Preclinical studies have shown that a single injection of ketamine immediately increases the firing and burst activity of NE neurons [75]. There are also relevant experimental studies showing that the firing activity of NE neurons is significantly enhanced after one day of repeated ketamine administration, while the increased activity of NE neurons is no longer present after three days of repeated ketamine injections [76].

### 3.3. Dopamine (DA)

DA is a catecholamine neurotransmitter synthesized from dietary amino acids [77]. The precursor of DA, L-DOPA (3, 4-dihydroxy-1-phenylalanine), is produced by TH catalysis of L-tyrosine [78]. Aryl amino acid decarboxylase (AAAD) is a rate-limiting enzyme that converts tyrosine to L-DOPA to DA through AAAD [79]. Clinical studies have found that most people with depression have low levels of DA, and it is thought that effects similar to antidepressants might be achieved by enhancing dopaminergic neurotransmission [80,81]. Dysfunction of the DA receptor and transporter is associated with neuropsychiatric diseases, including depression [82]. Studies have found that dopamine D1–D2 receptor isomers have positive effects on depressive and anxious behaviors [83]. This complex regulates the BDNF/TrkB signaling pathway and the Akt/GSK3/β-catenin signaling pathway, both important signaling pathways in antidepressant actions [84]. D1 receptor agonists can relieve behavioral depression associated with pain, although they have certain clinical limitations [85]. DA regulates depression-like behavior in mice through D2 receptors and plays a positive role in hippocampal learning and memory, providing evidence for DA involvement in neuropathy-related diseases that lead to mild cognitive impairment [86]. Related genetic evidence that D3R deficiency results in chronic depression and anxiety in rodents suggests that D3 receptors may be important mediators of depression, implying that D3R could be a target for designing more specific DA-based antidepressants [87]. Dysfunction of DA receptors and the transporter can induce many symptoms of neuropsychiatric diseases such as depression, although sometimes the opposite symptoms can occur; for instance, DA transporter knockout mice show reduced depression-like behavior [88]. Studies have shown that low doses of the partial DA agonist aripiprazole, a selective DRD2/DRD3 receptor agonist, can significantly improve depressive symptoms and may be a relatively safe adjunct treatment for major depression [89,90].

### 3.4. Glutamate (Glu)

Glu is synthesized from glutamine (Gln) and is a precursor for the synthesis of GABA. Glu can also be produced by transamination of α-ketoglutaric acid. Decreased levels of neurotransmitter Glu in mPFC and some other brain regions may be associated with depression [91]. Glu receptors are divided into metabolic glutamate (mGlu) and ionotropic glutamate (iGlu) receptors. The mGluR2/3 antagonist LY341495 activates the PI3K/Akt signaling pathway to stimulate the 5-HT1A receptor in the mPFC, leading to a sustained antidepressant-like effect [92]. Studies have suggested that the mGlu5 receptor antagonist 3-[(2-methyl-1,3-thiazol-4-yl)ethynyl]-pyridine (MTEP) has antidepressant effects and that the 5HT2A/2C antagonist ritanserin reverses the antidepressant-like effects of MTEP in TST [93]. Scopolamine is a muscarinic cholinergic receptor antagonist. It has been found that low-dose scopolamine combined with the mGlu7 receptor allosteric agonist AMN082, has potential antidepressant activity in C57BL/6 mice, which may be related to the activation of the mTOR pathway [94]. Models of chronic stress-induced depression provide evidence of glutamate–glutamine imbalances in the systemic circulation and the brain [95]. In mammals, by regulating glutamate + glutamine (Glx) metabolism, energy metabolism can be promoted, and depressive symptoms can be significantly improved [96]. The Glu ionotropic receptor subtype N-methyl-D-aspartic acid antagonist (NMDA) influences Glu excitatory neurotransmission by influencing amino-3-hydroxy-5-methyl-4-isothiazole propionic acid receptor (AMPAR) signaling [97]. Over the last 20 years, ketamine, an NMDA receptor antagonist, has shown antidepressant properties. The role of NMDA receptor regulation in the treatment of depression is that it drives synaptic and behavioral responses by blocking NMDAR on GABAergic interneurons and inhibiting glutamate bursts [98]. In one study, the hypothesized role of the glutaminergic system and the GABAergic system in antidepressant-like effects before, during, and after ketamine administration was evaluated by measuring Glx and GABA levels in the mPFC by proton magnetic resonance spectroscopy [99]. The study found that within minutes of ketamine administration, the levels of Glu in the mPFC of MDD patients increased rapidly [100].

### 3.5. Gamma-Aminobutyl Acid (GABA)

GABA has very broad effects on neural function, largely opposing the excitatory effects of Glu, and is directly or indirectly involved in the pathogenesis of many psychiatric diseases. GABA is synthesized by glutamate decarboxylase (GAD) via decarboxylation of Glu [101]. In animal experiments, the Chronic Unpredictable Mild Stress (CUMS)-induced depression-like model resulted in reduced GABA synthesis and release in the nucleus accumbens (NAc) tissue of mice, and clinical experiments also suggested that low GABA levels might be one of the pathogenic factors of depression [102,103]. Downregulation of GABA receptors triggered by astrocyte activation is a potential mechanism for early inflammation and an increased risk of depression in adulthood [104]. Activation of the GABAA and GABAB receptors produces antidepressant-like effects [105]. Brexanolone, one of the new generation of antidepressants, was developed to treat postpartum depression by targeting GABA receptors [106]. Oral administration of GABA can also regulate the decrease of monoamine neurotransmitters such as 5-HT in the HP in FST rats, thereby improving the symptoms of depression, which ties these mechanisms to longstanding theories of depression that involve monoamines [107]. The monoamine oxidase inhibitor phenylethylamine has antidepressant-like effects that increase the levels of GABA in the brain and have been shown to improve memory [108]. When endogenous synthesis is deficient, GABA can also be obtained through exogenous supplementation. Thus, a variety of types of findings suggest that the neurotransmitter GABA is closely associated with depression and increasing GABA levels can potentially prevent and improve depressive symptoms.

## 4. Other Hypotheses

### 4.1. Brain-Derived Neurotrophic Factor (BDNF)

BDNF is a neurotrophic protein in the brain that regulates various cellular processes by binding and activating its receptor, TrkB [109]. Studies have shown that reduced BDNF in the HP can induce depressive-like behavior and impair neuronal differentiation in the HP, supporting the neurotrophic hypothesis of depression [110]. Duman et al. first proposed that tricyclic and selective 5-HT reuptake inhibitor antidepressants increase BDNF expression in the rodent brain, which has been demonstrated in many studies [111,112]. In addition, ketamine, newly described as a so-called “fast-acting” antidepressant, can also increase hippocampal BDNF levels and enhance the remodeling of synapses associated with mnemonic improvement [113]. This mechanism has also been shown to be important in patients with depression in clinical trials. BDNF expression is increased in the HP of depressed patients treated with antidepressants [114]. The 5-HT gene knockout rat (SERT) model is one of the animal models of depression. Overexpression of BDNF in the ventral HP of SERT KO rats can reverse anhedonia and other depressive symptoms [115]. However, the study showed that BDNF-TrkB signaling plays a necessary role in mediating CSDS-induced social avoidance behavior in the ventral tegmental area (VTA)-NAc, and established BDNF-TrkB signaling as a pathological mechanism during chronic stress [116]. Moreover, electroconvulsive therapy (ECT) reduced VTA BDNF levels, and VTA BDNF knockdown alone induced an antidepressant-like effect, while VTA BDNF overexpression blocked the antidepressant-like effects of ECT [117]. These findings have indicated that the antidepressant effects of BDNF depend on the different brain regions.

### 4.2. Glial Cells

Stress contributes to the development of depression in part by changing the structure and function of astrocytes [118]. In mice experiencing acute emotional stress, GluA1 expression is regulated by the adrenergic receptor/adenylate cyclase/CPEB3 pathway, revealing a role of glial plasticity [119]. Electroacupuncture treatment produces antidepressant-like effects in rat models of depression induced by chronic stress by regulating hippocampal astrocyte atrophy [120]. Among the calorie restriction-induced antidepressant effects, astrocyte ATP in the mPFC may play an antidepressant role by affecting excitatory synaptic transmission [121]. Running could relieve the depressive symptoms after CUS in rats through its effects on astrocytes [122]. In addition, it was found that running exercise alleviated the depressive-like behavior of CUS in rats and positively affected the volume of the mPFC and protected mPFC and hippocampal oligodendrocytes [123,124]. The antidepressant-like effects of escitalopram involves BDNF and regulates the number of oligodendrocytes in the HP and frontal lobe [125]. Microglia are essential to the neuroimmune system. It has been suggested that microglia influence the development of depression, and that this relationship has a significant genetic basis [126]. Microglia signal transduction and transcription factor 3 (STAT3) regulate the synaptic function of macrophage colony-stimulating factor (M-CSF) in neurons that in turn that upregulate BDNF expression, which is central to its antidepressant role [127]. In addition, the downregulation of BDNF and TrkB in microglia can also lead to depressed behavior [128]. Chronic stress-induced depression-like and anxiety-like behavior were not only associated with microglia activation but also related to hippocampal neuroinflammation [129].

### 4.3. Inflammation Hypothesis

Inflammatory activation and the release of inflammatory cytokines plays an essential role in developing depression. Studies have shown increased mean levels of inflammatory markers in blood and cerebrospinal fluid (CSF) in MDD [130,131]. The Lipopolysaccharide (LPS) model is a mouse model of depression induced by inflammation [132]. The severity of the depression-like state in LPS-induced or LPS-plus stressed mice were positively correlated with the inflammatory response, and the change in depression-like behavior depended on the induction of proinflammatory cytokines [133]. Phenylalanine ammonia-lyase (PAL) can reverse depression-like behavior in the LPS model by balancing inflammatory and oxidative effects [134]. Inflammation is an important disease modifier that promotes susceptibility to depression, so controlling inflammation may provide an overall therapeutic benefit, whether secondary to early life trauma, a more acute stress response, or microbiome alterations [135]. In in vivo experiments, sodium hydrogen sulfide (NaHS) has been found to alleviate depressive-like and anxious-like behavior induced by type 1 diabetes mellitus (T1DM) by regulating the inflammatory response [136]. In addition, the antidepressant agomelatine has a neuroprotective effect and prevents apoptosis [137]. Research shows that a high-fat diet can cause oxidative stress in the CNS, leading to mood disorders and neuroinflammation [138]. All of these studies suggest that inflammation is involved in the pathophysiological processes of depression.

### 4.4. Neuroendocrine Systems

Depression is linked to HPA axis dysfunction. Corticosterone (CORT)-induced depression-like behavior is caused by oxidative stress due to abnormal activity of the HPA axis [139,140]. Certain antidepressants such as imipramine, mirtazapine have been reported to improve depressive behavior by regulating the HPA axis, [141,142,143]. Changes in intestinal flora that lead to depressive behavior and major neurochemical changes in mice may produce their effects via mechanisms involving the HPA axis, demonstrating connections between these systems [144,145]. Gut microbiota is an important part of the brain-gut axis, and changes in the microbiota affect brain development and the interaction between the gut and brain [146]. Trp is the only precursor of 5-HT and comes mainly from the diet [147]. Gut flora directly or indirectly regulates Trp metabolism and plays a key role in the pathophysiology of depression through the brain–gut axis [148]. Diet improves nervous and immune function through gut flora and regulates endocrine and metabolic systems through the brain–gut axis [149,150]. It was found that chronic stress in mice may participate in Trp metabolism and alter the intestinal microbiome through the gut–brain axis [151]. It was found that unpredictable chronic mild stress (UCMS)-induced depression-like behavior in rats is related to metabolomic changes in the brain–gut axis, the secondary effect of hippocampal neuroplasticity [152]. The intestinal microflora provides a new target for preventing and treating neuropsychiatric disorders [153]. Recent findings indicate that individuals diagnosed with Crohn’s disease or celiac disease, which can alter gut flora significantly, might be more prone to disturbances in the brain–gut axis [154,155]. These disruptions could potentially contribute to developing or exacerbating depressive symptoms in these patient populations [156,157]. Studies have indicated that approximately 25% of individuals diagnosed with Inflammatory Bowel Disease (IBD), including Crohn’s disease, may experience symptoms of depression [158]. In summary, the neuroendocrine system plays a pivotal role in the pathogenesis of depression, offering crucial insights into the multifactorial nature of this disorder.

## 5. Antidepressant-like Effects of Dietary Manipulations

**Figure 1:**
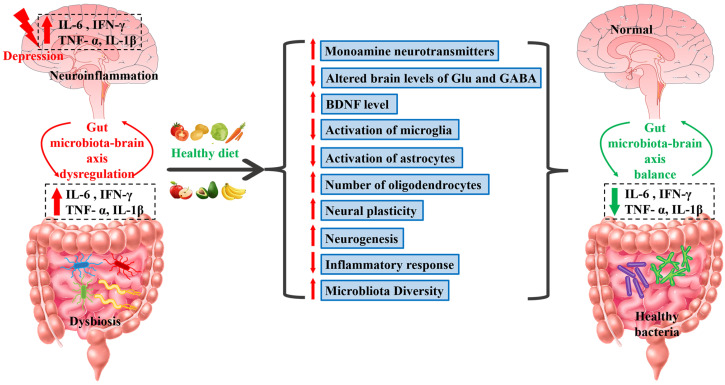
Summary of the relationship between gut microbial–brain axis and depression and the role of dietary regulation. The stress state leads to dysbiosis of the gut microbiota, increasing pro-inflammatory cytokines, mainly IL-6, IFN-γ, TNF-α, and IL-1β, and this situation can be reversed through dietary modulation. Dietary modulation may improve depressive symptoms by upregulating monoamine neurotransmitters, modulating the levels of glutamate and GABA in the brain, upregulating BDNF expression, modulating glial cells, synaptic plasticity, and neurogenesis, as well as reducing the inflammatory response and the diversity of microbiota. IL-6: interleukin-6; IFN-γ: interferon gamma; TNF-α: tumor necrosis factor; IL-1β: interleukin-1β. Upward arrows: increase. Downward arrows: decrease.

Food provides the human body with nutrients for growth, development, and healthy survival [159]. In Figure 1, we summarize the relationship between the various mechanisms of depression pathogenesis mentioned in the previous chapter and dietary modulation. Dietary intake of nutrients involves a complex system that includes complex interactions between diet, gut microbiome, and energy metabolism [160]. After ingestion, food is gradually reduced to smaller molecules and absorbed, primarily in the intestine [161]. During absorption, molecular species increase significantly as larger polymeric molecules are reduced to simpler ones, including proteins, carbohydrates, and others [162]. In the intestinal tract, proteases with different substrate specificities degrade proteins to produce peptides with different amino acid chain lengths and sequences [163]. Enzymolysis is used in the gastrointestinal tract and microorganisms to search for bioactive peptides produced from food proteins [164]. This process involves an interaction between the microbiome and the host, and numerous signals move back and forth between each, substantially impacting host function [165,166]. These interactions affect psychological and physiological function, and it appears that proper nutrition can strengthen the immune system with substantial effects on mental health, including depression [167]. Dietary therapy uses food to affect the function of various aspects of the organism so that it can achieve a treatment or prevent diseases [168,169,170,171,172,173]. Table 2 summarizes the antidepressant effects of various foods. Dietary interventions are considered cost-effective potential treatment options for depression [174]. While there are no antidepressant foods per se, food can improve depressive symptoms through several intervention mechanisms associated with healthy eating habits [175]. Table 3 summarizes the active ingredients of some representative foods and their mechanisms of antidepressant action. This includes regulating the function of neurotransmitters to improve mood. While such interventions are unlikely to replace drugs in many cases, they can limit certain toxicity and side effects, offering a new way to treat mild depression or as adjuvant therapy [176]. This treatment may involve various intervening factors that contribute to the development of depression. For instance, obesity is highly co-morbid with depression, which can lead to a vicious cycle of emotional eating that further exacerbates metabolic impairments [177]. The brain–gut axis is an intervention point for obesity and treating mood disorders involving the CNS [178]. Studies have shown that gut flora alters central nervous system function and is involved in the pathogenesis of depression [179]. There is evidence of an interaction between the microbiome in the brain and inflammasome activation and that the immune system may be involved in regulating the balance between the brain and the gut [179,180]. Stressful situations can result in gut microbiota dysbiosis and an elevation in pro-inflammatory cytokines (IL-6, IFN-γ, TNF-α and IL-1β) [180]. Intestinal microbial imbalance can affect the function of the HPA axis, leading to systemic inflammation and immune dysfunction. Improving intestinal dysregulation may be a potential treatment for depression [181,182,183]. (Figure 1).

### 5.1. Fruits and Vegetables

A diet abundant in fruits and vegetables enhances various dimensions of health and well-being, encompassing mental health. Głąbska et al. conducted a comprehensive review summarizing the repercussions of decreased consumption of fruits and vegetables. Reduced intake might lead to depression-like symptoms, whereas increased consumption of these foods has been associated with enhanced mood, improved sleep, cognitive function, and overall quality of life [227]. Research indicates that consuming a diet abundant in fruits and vegetables can notably enhance general well-being, encompassing cardiovascular health [228]. Achieving this impact on overall mental well-being necessitates daily consumption of a minimum of five servings of fruits or vegetables [229,230]. Broad physiological and metabolic effects may mediate these effects. Various physiological effects arise from differences in fruit consumption, including anti-inflammatory and antidepressant-like effects [231]. Although the effects above may involve quite general mechanisms, specific substances in certain types of foods may contribute to their effects. Bananas are aromatic, nutritious, widely available, and can be harvested throughout the year. Banana stem extract has significant antidepressant activity in forced swimming, including animal models of depression and regulates neurotransmitters, so it is a potential natural compound used to treat depression [232]. Furthermore, analyzing the content of banana pulp at various developmental stages, unveiled its abundance in tryptophan, potentially attributing to these effects [233]. Pomegranate is known for its high nutritional value. Pomegranate is rich in estrogen and shows estrogenic activity in mice [234]. It was found that pomegranate extract alleviated depressive behavior in ovariectomy-stimulated depression model mice by enhancing the central adrenergic system and inhibiting ovariectomy-stimulated bone turnover [235]. The liquid extract of pomegranate regulates the activation of the estrogen receptor and serotonergic system, has an antidepressant effect, is advantageous in the treatment of menopause, and has an additive effect when combined with a selective 5-HT reuptake inhibitor [185]. Citrus fruits and vegetables are rich in quercetin, which has anti-inflammatory and antioxidant properties [236]. Quercetin inhibits the activation of the NLR family, Pyrin Domain containing three (NLRP3) inflammasome by promoting mitotic phagocytosis and thereby reducing neurotoxicity [237]. A long-term high-quercetin diet can inhibit the activation of astrocytes that have pro-depressive effects and protect neurons [186]. It has been reported that quercetin and its derivatives have the antidepressant potential of protecting hippocampal neurons, improving HPA axis dysfunction, and improving neuroplasticity [204]. However, because of the multi-target and multi-pathway characteristics of quercetin and its derivatives, it is significant to explore its anti-depression effect and related mechanisms and promote its clinical application. Vegetable soybean nutrition is comprehensive, rich in protein, and highly nutritious. It may help build the human body’s immunity and prevent lifestyle-related diseases [238]. Genistein, a natural isoflavone found in soybean extract, has been shown to have antidepressant-like effects through FST and TST, and its antidepressant-like effects may be due to increased levels of 5-HT in the mouse brain [187]. One study reported that high-pressure processing technology could increase the content of GABA in vegetable soybeans and that GABA-rich vegetable soybeans not only prevent depression but also significantly reduce depressive symptoms in UCMS mice [239]. Perilla frutescens is a side dish in East Asian countries and a plant-based medicine. Perilla frutescens can reverse the depression-like behavior in CUMS mice, and its antidepressant activity may be related to the changes in serotonergic and anti-inflammatory effects [240]. In addition, research has indicated that frequent consumption of fried foods, such as fried potatoes, is associated with an increased risk of anxiety and depression, closely related to lipid metabolism dysfunction and neuroinflammation mediated by acrylamide, a representative pollutant in fried foods [241]. These findings suggest that dietary patterns characterized by healthy fruits and vegetables may lower the risk of depression.

### 5.2. Fish

The high levels of omega-3 polyunsaturated fatty acids (PUFA) in fish may lead to their physiological effects, and it is clear that the increased consumption of fish relative to other protein sources has a large impact on human health [242]. Regular fish consumption in the elderly is also linked to a reduced risk of depression later in life, which may be linked to a higher intake of omega-3 PUFA [243]. Most recently, researchers have found that taking omega-3 supplements during COVID-19 infection can improve symptoms such as depression associated with long COVID [244]. In animal studies, omega-3 PUFA supplements can reduce brain injury and play a neural protective role in rats with trauma [245]. In addition, omega-3 PUFA has demonstrated a positive effect on sleep, cognition, depression, and anxiety in sleep-deprived rats [246]. An increase in the omega-6/omega-3 fatty acid ratio has been reported to increase the risk of obesity, which affects inflammatory status and is co-morbid with mental health issues [247]. Dietary omega-3 PUFA reduces clinical colitis by reducing pro-inflammatory cytokine synthesis and improving epithelial barrier function in animal models [248]. Fish oil mainly consists of omega-3 PUFA, including docosahexaenoic acid (DHA) and eicosapentaenoic acid (EPA). Fish oil supplements can improve the mental health of patients with depression [249]. In animal studies, fish oil intake significantly increases concentrations of DHA and EPA in rat HP and cerebral cortex, which may be associated with antidepressant-like effects [250]. Dietary DHA supplements may also improve depression by regulating gut microbes [251]. DHA helps restore immune function in the microbiome, reduces stress-induced inflammatory responses, and prevents overactivation of the HPA axis [252]. Epidemiological reports suggest that these effects of omega-3 PUFA may normalize feedback control of the neuroendocrine HPA axis and increase corticosterone transport [253]. Omega-3 PUFA activates the HPA axis by increasing cortisol secretion [254]. Omega-3 PUFA preconditioning corrects LPS-induced depressive responses, which involve the secretion of pro-inflammatory cytokines, changes in intestinal structure, and alterations in the intestinal flora [255]. According to the report, a diet rich in omega-3PUFA plays an antidepressant role by increasing the level of 3, 4-dihydroxyphenylacetic acid (DOPAC) in mouse NAc and the number of TH-positive neurons in VTA, suggesting that the mechanism may be related to the regulation of the NAc-VTA dopaminergic signaling [256]. Some studies have observed changes in neural behavior after treatment with dietary fish oil and found that fish oil has anti-anxiety and anti-depressive potential effects [257]. During pregnancy and lactation, fish oil intake has been shown to reduce the likelihood of the development of depression-like behavior in offspring [258]. In a depression-like model of ovariectomized (OVX) rats, omega-3 PUFA supplements have antidepressant-like effects, partly by protecting nerves by regulating inflammatory cytokines and interfering with microglial polarization [259]. Caloric restriction and the fish oil diet promoted anti-anxiety and improved memory, and these effects were correlated with BDNF concentrations [260]. A comparison of heterozygous BDNF mice with wild-type BDNF showed that long-term fish oil supplementation restored extracellular 5-HT levels by increasing 5-HT transporter expression [189]. In particular, fish oil supplements may have antidepressant-like effects in the HP through activation of the 5-HT1A receptor and enhanced 5-HT neurotransmission [261]. Further research is needed to determine whether fish oil supplements can be alternative and complementary medicine to improve the symptoms of depression. Studies have found that liquid extracts from fish fillets may mediate antidepressant effects through 5-HT and NE systems [190]. In addition, omega-3 PUFA has been shown to have a positive effect in regulating microbial composition, helping to improve cognitive function in maternal depression and other animal models of depression [262]. Omega-3 PUFA has a modest beneficial effect on depressive symptoms, and future studies are needed to prove the potential advantages and disadvantages of omega-3 PUFA on depression [263].

### 5.3. Drinks

Caffeine, a widely consumed psychoactive substance in beverages such as Coca-Cola^TM^ (Coke), tea, and coffee, exerts mild activating and mood-enhancing effects. Functional beverages crafted from various components possessing potential antidepressant properties exhibit notable antidepressant effects by enhancing the monoamine neurotransmitter system and exerting antioxidant effects [264]. In rats receiving CUS, long-term caffeine use has been shown to affect depression-like behavior, including withdrawal symptoms typical of other drug abuse, accompanied by increased levels of DA and 5-HT, suggesting that the dopaminergic and serotonergic systems may be involved [191]. Caffeine treatment can restore social avoidance and anhedonia induced by social failure stress in mice through dopaminergic system regulation, confirming the potential therapeutic value of caffeine [265]. There is evidence that combining caffeine and some antidepressants may affect Glu, adenosine and monoamine systems associated with depression, perhaps in an additive manner to reverse depressive pathophysiology [266]. When caffeine is combined with duloxetine or bupropion, the antidepressant-like effects are significantly enhanced, especially in the cerebral cortex, where NE, DA, and 5-HT levels are significantly increased [267]. Caffeine increases extracellular 5-HT levels and inhibits 5-HT reuptake, thereby elevating 5-HT function [268]. The direct pharmacological effects of caffeine occur mainly through adenosine receptor antagonism, preventing adenosine from inhibiting the release of NE, which would also be expected to play a positive role in the treatment of depression [269]. Combined administration of caffeine and NMDA receptor antagonists (e.g., fast-acting antidepressants) enhances antidepressant-like effects [270]. Therefore, synergistic antagonism of adenosine and NMDA receptors may offer a novel way to improve depression therapy. Of course, excessive caffeine consumption is not advisable and may cause cognitive decline, depression, insomnia, and other symptoms [271]. People who regularly consume soda or processed juices also have a higher risk of depression [272]. In conclusion, as a psychostimulant, more prospective studies are needed to determine whether caffeine consumption decreases depressive symptoms. Other active ingredients in tea (L-theanine, L-arginine, polyphenols) and their metabolites act together in multiple biological systems to reduce the risk of depression [273]. The various active compounds in tea can play an antidepressant role and reduce potential biological toxicity by improving HPA axis dysfunction, anti-inflammatory effect, restoring the monoaminergic system, improving intestinal flora, and promoting gut–brain axis activity [274]. Tea in moderation is also beneficial for a range of cardiovascular diseases [275]. Tea with a GABA content of 1.5 mg/g total weight or more is called GABA green tea. An animal study showed that GABA green tea might promote the expression of the α1 subunit of the GABA receptor in the cerebral cortex of mice and improve depressive symptoms by upregulating the expression of GABA [276]. Green tea polyphenols significantly reduced the immobile time of FST and TST in mice, and the antidepressant-like mechanism of action may involve the HPA axis [277]. Therefore, green tea may have the effect of preventing and alleviating depression. Alcohol as a drinking relationship with depression is complex. Anxiety is often accompanied by alcoholism and can aggravate depression [278]. However, in an animal study, 0.7 g/kg ethanol was found to have an antidepressant-like effect in genetically selected alcohol-eligible rats [279]. Clinical studies have found that 5 to 15 g of alcohol intake per day, especially wine intake, may reduce the incidence of depression [280]. Alcohol is similar to the antidepressant ketamine in regulating dopaminergic and glutaminergic pathways in the limbic cortex and may mediate antidepressant therapy [281]. One study found that mice transplanted with feces from people with alcohol use disorders (AUD) showed increased depression-like behavior, suggesting that intestinal microbiome disturbances, neurotransmission, and neuroinflammation may be associated with alcohol addiction [282]. Excessive drinking is not advisable and may pose a threat to health. Probiotics refer to bacteria usually found in fermented foods such as yogurt or beer, which confer health benefits. Clinical microbial therapies, such as probiotics, may be cautiously recommended to patients with depression to promote good bacteria in the gut and improve mood through the gut–brain axis [283]. Indeed, one study has shown that probiotic-containing functional wheat beer (PWB) promoted antidepressant-like effects in Swiss Webster mice [284]. Kefir peptide (KP) is a novel antidepressant dairy product that improves depressive-like behavior by activating the BDNF/TrkB pathway [285]. Clinical studies have shown that probiotics can improve mental illness-related behavior by improving changes in the gut microbiome [286]. Related studies have demonstrated the protective effect of probiotics on depression-like behavior in high-fat diet (HFD)-induced FSL rats [287]. Plasma metabolites may reveal a relationship between abnormal microbiome function and depression and the consequent antidepressant effects of probiotics [288]. FSL rats exhibit elevated plasma concentrations of NE and DA. Notably, probiotic intervention can mitigate the impact of plasma NE and DA on depressive states [289]. Although the pharmacological effects of probiotics need further study, they appear to interact with many well-known antidepressant mechanisms [290]. Furthermore, this prompts the inquiry of whether the converse holds true, and whether certain antidepressant actions entail direct influences on the intestinal microbiota. Therefore, additional investigation is warranted into the pharmacological impacts of probiotics.

### 5.4. Vitamins

Vitamins are essential dietary components for life and are crucial to health. Vitamins B1, B3, B6, B9 and B12 are vital for neuronal function, and a lack of B vitamins may contribute to the development of depression [291]. Vitamin B deficiency can lead to central and peripheral nervous system abnormalities and accompany some psychiatric disorders [292]. One study suggested that vitamin B supplementation can reduce environmental factor induced depressive behavior in mouse offspring during pregnancy, which is related to the inhibition of apoptosis, oxidative damage and inflammation [293]. Vitamin B6 may also affect depression by altering glucocorticoids [294]. Folic acid is a vitamin B complex with many metabolic effects. Green vegetables, animal livers, yeast and radishes are good sources of folic acid [295]. The body cannot produce folic acid, so it needs to be taken from food. Studies of depressed patients have found low levels of both folic acid and vitamin B12, and studies of the general population have found an association between depression and low levels of both vitamins [296]. Animal studies have found that the interaction between the serotonergic and NE systems may be responsible for the antidepressant-like effects of folic acid [194,297]. Folic acid increased brain and serum BDNF levels in CUMS rats, which could be used as a potential antidepressant [194]. Folic acid also reverses depressive behavioral changes induced by corticosterone [298]. When the antidepressant venlafaxine was combined with folic acid, the therapeutic effect increased, which was associated with synergistic effects on 5-HT levels [299]. Vitamin C can enhance the body’s immunity [300]. Fruits such as citrus and pomegranates are rich in vitamin C. Animal studies found that adding lemons and pomegranates to the diet reduced anxiety and depression in rats [301]. The antidepressant-like effects of Vitamin C administration in the TST are associated with effects on 5-HT, NE, and the dopaminergic systems [302]. In addition, studies have suggested that the antidepressant-like effect of vitamin C in the TST is related to the activation of the GABAA receptors and antagonism of the GABAB receptors in the dopaminergic system [303]. The protective effect of vitamin C in neurological diseases involves increased levels of NE and GABA, and the antidepressant-like effects are similar to ketamine [304,305]. Vitamin D also appears to improve depression-like behavior in animal models, including the CUMS and OVX models, through actions on BDNF and neurotrophic protein (NT)-3/NT-4 signaling pathways and serum corticosterone/adrenal corticotropin (ACTH) levels [197]. There is also evidence that vitamin D may be used as a supplement for treating depression in older people [306]. Vitamin D deficiency in older adults leads to bone mineralization disorders and increases the risk of cardiovascular disease and depression [307]. However, excessive vitamin D intake can interfere with phosphate homeostasis regulation and affect health, so it is recommended to increase vitamin D intake reasonably [308]. These findings imply that vitamin supplementation may enhance mood, although additional research is required to ascertain the underlying mechanisms in greater detail.

### 5.5. Homology of Medicine and Food

Many foods or food flavorings may also have antidepressant-like effects in addition to other potential medical benefits [309,310,311,312,313]. These include ginseng, turmeric, piperine (PIP), saffron and many others. The molecular and cellular mechanisms of ginseng and its active ingredients regulate monoamine neurotransmitters, upregulate the expression of BDNF and have anti-inflammatory effects, which are of great importance in treating depression [314]. Panax ginseng extract improves depression-like behavior in rats primarily by promoting hippocampal neurogenesis and the BDNF-TrkB signaling pathway [315]. The antidepressant mechanism of ginsenoside Rb1 is primarily through activating the neurotransmitter in 5-HT, NE, and DA systems [198]. Moreover, evidence supports the importance of 5-HT2A receptors in ginsenoside Rb1-induced antidepressant mechanisms [316]. Curcumin (CUR) is one of the main active components in turmeric, which can be used as medicine and food. Studies have shown that demonstrated CUR has antidepressant activity, possibly due to elevated levels of 5-HT, NE, and DA [317]. CUR can improve depressive symptoms in OVX mice, in part by regulating the balance of 5-HT1A and 5-HT2A receptors [318]. CUR derivatives also have antidepressant activity, activating 5-HT1A receptors and Camp/PKA/CREB/BDNF signaling pathways to produce therapeutic effects [319]. In addition, CUR may achieve antidepressant effects by upregulation of BDNF, reducing inflammatory factors in the brain, and enhancing cell activity and synaptic plasticity, all mechanisms shared with traditional antidepressants [199]. PIP, the main ingredient in pepper, is also an anticonvulsant. Indeed, a wide range of PIP derivatives have different biological effects and the potential to treat and prevent disease [320]. Intraperitoneal injection of PIP has shown that it mediates antidepressant-like effects by increasing 5-HT content in mice brains [200]. Further studies are needed to verify the interaction between the 5-HT receptor and PIP. Chronic PIP therapy exerts antidepressant-like effects by increasing BDNF levels in the mPFC of CUMS mice [201]. Saffron is a traditional Chinese medicine that also is a common food flavoring. Oral administration of saffron increased serum DA and CREB levels and improved depression-like behavior through intestinal flora in mice exposed to chronic restraint stress [202]. Schisandra Chinensis and its active constituents have protective effects on neurological diseases [321]. In FST-depressed mice, Schisandra extract may mediate antidepressant effects through NE, DA, GABA and Glu systems [203]. Schisandra extract may ameliorate the depression-like emotional state and related cognitive deficits in CUMS mice by mediating the level of BDNF in the HP [322]. Raw and wine-processed Schisandra chinensis may improve depression-like behavior in chronic unpredictable stress program (CUSP) rats by regulating intestinal flora [323]. These findings suggest that various common flavorings also have some potential pharmacological effects, including antidepressant-like effects, although the effectiveness of these drugs in patients with depression remains to be demonstrated.

### 5.6. Other Foods

During FST experiments, oral administration of chicken breast extract or carnosine to male Wistar rats influenced quiescent time and notably elevated hippocampal NE concentration [324]. A study has found that a healthy diet of non-processed meats such as beef and lamb may reduce the risk of depression [325]. Moreover, the beef protein diet-induced elevated 5-HT release in the brains of mice, indicating the activation of the 5-HT system [326]. Clinical studies have found a positive correlation between the frequency of processed meat and animal offal consumption and the risk of depression [327]. While the link between the mechanisms underlying the antidepressant-like effects of meat consumption and neurotransmitter action remains unestablished, the observed antidepressant effects resulting from changes in meat intake—likely influenced by underlying nutrient content—pave the way for future research. Sesamin, found in sesame seeds, is a kind of lignan. In animal studies, long-term use of sesamin has a neuroprotective effect and can prevent brain dysfunction through antioxidant activity [328]. In CMS-induced depressed mice, sesamin can prevent stress-induced mood disturbances and inhibit the inflammatory response of CMS [329]. It prevents stress-induced reduction in 5-HT and NE in the striatum and serum, thereby significantly improving depression-like behavior and anxiety in mice with CUMS [330]. Sesamin as a food component may be a potential new treatment for depression. A diet rich in seed oil had antidepressant effects and increased BDNF levels in the rats’ brains during FST and TST [331]. The study suggests that the potential antidepressant-like effects of sunflower consumption in CUMS-induced mouse models of depression may arise from augmented concentrations of aromatic amino acids and monoamine neurotransmitters, including 5-HT, DA, and NE [332]. Dairy products have many benefits for human health. Recent studies have found that GABA-rich functional fermented milk has significant antidepressant-like effects, suggesting that GABA-containing dairy products can be used as a new dietary therapy to exert antidepressant-like effects [333,334]. Gouda cheese induced alterations in the microbiome of CUMS mice, contributing to the restoration of cognition and enhancement of mood and brain function [335]. A clinical study from Japan found that the frequency of consumption of low-fat dairy products was inversely associated with the risk of depressive symptoms [336]. Additionally, the consumption of skim milk and moderate quantities of dairy desserts exhibited a negative correlation with depressive symptoms, whereas the consumption of whole milk demonstrated a positive correlation with depressive symptoms in adults [337]. Interestingly, another study found that high-fat and low-fat dairy products were linked to a reduced incidence of psychological disorders [338]. The possible mechanism of dairy products in depression is unclear, and more prospective studies are needed.

### 5.7. Dietary Treatments for Depression

**Figure 2:**
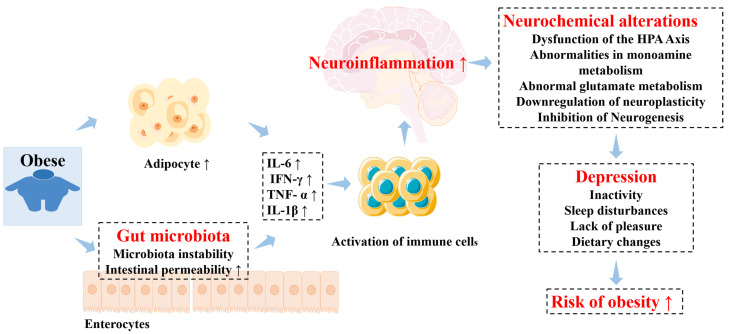
The relationship between obesity, inflammation, and depression. IL-6: interleukin-6; IFN-γ: interferon gamma; TNF-α: tumor necrosis factor; IL-1β: interleukin-1β. HPA Axis: Hypothalamic–Pituitary–Adrenal Axis. Black up arrows: increased correlation. Blue arrows: causation/induction.

The Expert Consensus of Overweight/Obesity Medical Nutrition Therapy in China (2016 edition) advocates 5 days of normal eating and 2 days of light fasting, called 5 + 2 light fasting, which can intervene in bad dietary habits and improve mood [339]. The Orthodox Christian Church (COC) dietary regimen involves strict fasting periods characterized by the abstention from animal products and the increased consumption of fruits, vegetables, and legumes. The COC fasting diet is associated with lower levels of depression and anxiety and better cognitive performance in middle age [340]. However, studies have found that a long-term low-quality diet can increase the likelihood of depressive symptoms in elderly individuals [341]. For older persons, adopting a healthy “Mediterranean” diet rich in vegetables and whole grains may reduce the risk of developing depressive symptoms [342]. Population studies suggest that dietary patterns and specific dietary factors may reduce the risk of depression [173]. The results of a meta-analysis suggest that a healthy diet of fruits, vegetables, whole grains, and fish may reduce the risk of depression, while a Western diet characterized by processed meat, refined grains, sweets, and high-fat dairy products may increase the risk [343]. Furthermore, it is worth considering the potential links between diet-related factors, such as obesity and metabolic conditions such as type 2 diabetes, and their impact on mood and mental health [344,345]. Animal studies have provided valuable insights, demonstrating that a short-term high-fat diet can lead to the overactivity of orexin neurons, potentially contributing to persistent depressive states [346]. Additionally, this dietary factor induces anxiety and anhedonia in rats by altering the insulin/mTORC1 signaling pathway, affecting synaptic plasticity and the production of pro-inflammatory cytokines [347]. The significant comorbidity between obesity and depression, which can lead to a vicious cycle of emotional overeating exacerbating metabolic dysfunction, highlights the complex relationship between metabolic and mood disorders [348]. Additionally, there is a correlation between obesity and chronic low-grade inflammation, which may directly contribute to depression in individuals with obesity [349,350]. The complexity of this interaction is further underscored by the potential of obesity to trigger both an increase in fat cells, leading to the release of inflammatory factors, and instability in the gut microbiota, which also contributes to the release of inflammatory molecules [351,352]. Activation of inflammatory factors stimulates immune cells, resulting in neuroinflammation and subsequent neurochemical changes, including HPA axis dysfunction, neurotransmitter metabolism abnormalities, and neurotrophic factor disruptions [353,354]. These alterations may contribute to depression development and increase the risk of obesity [355]. These intricate relationships are visually illustrated in Figure 2. Additionally, our laboratory’s research has revealed intriguing insights into dietary conditioning, including a 9-h fasting regimen, which demonstrates an antidepressant-like effect and significantly improves depression-like behavior in OVX mice [356]. The possible mechanisms may be related to the activation of the 5-HT2 receptor and CREB-BDNF pathway [356,357]. Dietary interventions sometimes produce conflicting results in studies, and this complexity needs further study [358]. These results underscore the importance of making healthy dietary choices. There is no perfect food, only a proper diet.

## 6. Limitations

The majority of studies encompassed in this review pertain to preclinical animal trials. Nevertheless, a scarcity of clinical literature exists concerning dietary interventions for preventing and managing depression. Challenges emerge when attempting to extrapolate findings from animal studies to clinical contexts, underscoring the necessity of enhancing the alignment, timing, and scope of clinical investigations. The foundation established by animal studies thus guides the direction for future clinical research. In clinical research, efforts must be directed toward discerning the specific constituents of healthy dietary interventions. This entails delving into the potential synergies among various food elements to mitigate the prevalence of depression. Such research promises to unveil efficacious therapeutic avenues for both clinical practice and public health settings. By delving into the intricate interplay between dietary factors and depressive states, we can shape a more comprehensive understanding of the impacts of diet on mental well-being.

## 7. Overview

**Figure 3:**
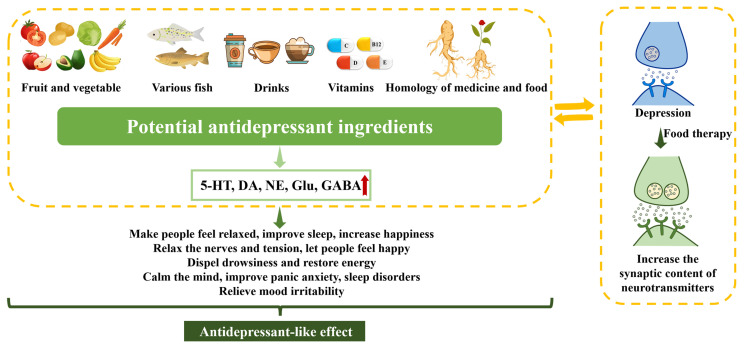
Summary of antidepressant effects of typical foods and their effects on neurotransmitters. GABA: Gamma-aminobutyl acid; 5-HT: Serotonin; DA: dopamine; NE: Norepinephrine; Glu: Glutamate. Red up arrows: increased correlation. Green arrows: causation/induction.

In this review, we summarized the antidepressant-like effects of typical foods and their effects on neurotransmitters (Figure 3). Antidepressant-like effects are seen in a wide range of foods, including vegetables, fish, caffeine, vitamins, and meat. The antidepressant mechanisms of food mainly involve monoamine neurotransmitters (5-HT, DA, NE), BDNF, glial cells, inflammation, neuroendocrine and so forth, mechanisms that are also seen in pharmacological treatments. Dietary treatment has become a trend to prevent and improve depressive symptoms, either in a preventative fashion or as a supplement to other treatments. The precise mechanisms remain to be fully elucidated, as do the effect magnitudes of dietary changes, but such interventions would have many obvious benefits. Changes in eating, such as reduced appetite loss or overeating, represent significant symptoms of severe depression. Hence, the potential influence of diet on depression is not unexpected. The emerging field of food therapy holds promise as a novel avenue for treatment development, offering a potentially lower toxic potential for adverse side effects than traditional pharmacological approaches. To advance our understanding and therapeutic strategies, further research is warranted to explore the intricate relationship between dietary intake and depression. Additionally, investigating potential interactions between diet and medications could provide valuable insights to bolster efforts aimed at preventing and improving the treatment of depression.

## 8. Future Perspectives

While we have summarized the potential effects of foods on combating depression and the associated mechanisms, several important avenues for future investigation emerge. Future research should delve deeper into the mechanisms of different food components on neurotransmitters, brain-derived neurotrophic factors, and other depression-related biomarkers. Clinical studies can illuminate how diverse populations might benefit from dietary interventions and effective methods for integrating such treatments into clinical practice. Additionally, investigating potential interactions between food and medication is crucial for comprehensively understanding treatment outcomes. Tailoring dietary intervention plans for individual patients based on their unique physiological characteristics and dietary habits may be an exciting area for exploration in future research. Discussing these future perspectives emphasizes the potential of this field and can inspire additional research efforts to explore the therapeutic potential of food in addressing depression.

## Figures and Tables

**Table 1:** Mechanisms of action and common side effects of antidepressant medications.

NO.	Antidepressant Class	Representative Medications	Putative Antidepressant Mechanisms	Common Side Effects	Limitations	References
1	SSRIs	Paroxetine, sertraline, and fluoxetine	Enhancement of synaptic 5-HT levels via reuptake inhibition	Gastrointestinal disturbances, insomnia, impaired memory, and sexual dysfunction	Potential risk of suicidality in individuals aged 18–24	[10,11]
2	SNRIs	Venlafaxine and duloxetine	Inhibition of 5-HT and NE reuptake, increasing their levels in the synaptic cleft	Gastrointestinal symptoms, sleep disturbances, and sexual dysfunction	Potential dependence issues during extended use	[12,13,14]
3	SARIs	Trazodone and nefazodone	Inhibition of 5-HT reuptake and antagonism of 5-HT2A receptors, resulting in elevated synaptic 5-HT levels	Dizziness, constipation, and drowsiness	Dose-dependency and substantial individual variability	[15,16]
4	MAOIs	Phenelzine and isocarboxazid	Inhibition of monoamine oxidase, leading to increased concentrations of neurotransmitters (5-HT, NE, and DA) in the brain synapses	Gastrointestinal reactions, dizziness, insomnia, orthostatic hypotension	Due to tyramine sensitivity, dietary restrictions required; potential drug interactions necessitate blood pressure monitoring	[17,18]
5	TCAs	Amitriptyline and doxepin	Elevation of 5-HT and NE levels in the synaptic cleft	Weight gain, constipation, dizziness, and cardiac side effects	Due to its elevated anticholinergic side effects and propensity for arrhythmias, it may not be appropriate for certain individuals with heart conditions	[19,20]
6	AAPs	Olanzapine and quetiapine	DA and 5-HT receptor antagonism, and modulation of other neurotransmitters	Weight gain and metabolic disturbances	Potential for obesity and related health issues	[21]
7	NaSSAs	Mirtazapine	Enhancement of NE release and 5-HT synaptic concentrations	Weight gain, dry mouth, and drowsiness	Potential for obesity and related health issues	[22]
8	NDRIs	Bupropion	Inhibition of NE and DA reuptake, leading to increased synaptic concentrations	Headache, insomnia, nausea, and loss of appetite	Occurrence of low tolerance or allergic reactions in some patient	[23,24,25]

Note: SSRI: Selective Serotonin Reuptake Inhibitors; 5-HT: 5-Hydroxytryptamine; SNRI: Serotonin-Norepinephrine Reuptake Inhibitors; NE: Norepinephrine; SARI: Serotonin Antagonists and Reuptake Inhibitors; MAOI: Monoamine Oxidase Inhibitors; DA: Dopamine; TCA: Tricyclic Antidepressants; AAP: Antidepressants and Antipsychotic Medications. NaSSA: Noradrenergic and Specific Serotonergic Antidepressants; NDRI: Norepinephrine and Dopamine Reuptake Inhibitors.

**Table 2:** A variety of foods produce antidepressant-like effects in depression models.

NO.	Food Type	Food Name	Subjects	Depression Model	Ingredients	Period	Putative Antidepressant Mechanisms	References
1	Fruit	Bananas	Locally bred albino Wistar mice	FST	Banana fruit pulp and peel	14 days	Increase in antioxidant enzymes (e.g., CAT, SOD) levels and reduction in GSH levels	[184]
2	Fruit	Pomegranates	Female Wistar mice	OVX	Pomegranate extract	14 days	Enhancement of central adrenergic function; activation of estrogen receptors and serotonergic systems	[185]
3	Fruit	Apple peels and citrus fruits	Male wildtype mice	CSDS	Rich in quercetin	30 days	Inhibition of astrocyte activation and neuroprotection	[186]
4	Vegetable	Soybean	Male ICR mice	FST, TST	Genistein	3 weeks	Regulation of brain 5-HT levels	[187]
5	Fish	Fish oil	Male Sprague–Dawley rats	LPS	ω-3PUFA	21 days	Inhibition of activation of inflammatory NLRP3 and ionic purine receptor P2 × 7R	[188]
6	Fish	Fish oil	Mixed S129/Sv x C57BL/6 genetic mice	BDNF +/− mice and their wild-type	ω-3PUFA	3 months	Reduction in hippocampal extracellular 5-HT levels and increase in Erk activation	[189]
7	Fish	Fish fillets	Male ICR mice	TST	AECSF	4 consecutive days	Regulation of serotonergic and norepinephrine systems	[190]
8	Drinks	Coke or coffee	Male Wistar rats	CUS	Caffeine	4 weeks	Increase in DA and serotonin levels	[191]
9	Drinks	Yogurt or beer	Male adult FSL and FRL rats	FSL	Probiotics	9-week period	Normalization of microbiome function	[192]
10	Vitamins	Vitamin B	C57BL/6 mice of either sex	CMS	VB12	10 weeks	Reversion of NTRK-2 gene expression	[193]
11	Vitamins	Vitamin B	Male SD rats	CUMS	Folic acid	6 weeks	Increase in monoamine neurotransmitter levels, BDNF, β-endorphins, and interleukin 6	[194]
12	Vitamins	Vitamin C	Adult Swiss mice of either sex	FST, TST	Ascorbic acid	1 day	Interaction of ascorbic acid with monoaminergic system	[195]
13	Vitamins	Vitamin D	Male C57BL/6 mice	PSD	VD3	4 weeks	Upregulation of the expression of BDNF in the HP	[196]
14	Vitamins	Vitamin D	Adult female Wistar rats	CUMS and OVX	VD3	28 days and 3 months	Reduction in serum corticosterone/ACTH levels; the increase in BDNF and NT-3/NT-4 levels	[197]
15	Homology of medicine and food	Ginseng	Male ICR mice and Wistar rats	CUMS	Ginsenoside Rb1	21 days	Activation of 5-HT, NE, and DA systems	[198]
16	Homology of medicine and food	Curcumin	Male SD rats	Chronically Stressed Rats	Diarylheptanoid component	18 days	Upregulation of BDNF and decreased brain inflammatory factors	[199]
17	Homology of medicine and food	Piperine	Male ICR mice	CUMS	Major alkaloids of black pepper and long pepper	3 weeks	Enhancement of 5-HT content and BDNF protein expression	[200,201]
18	Homology of medicine and food	Saffron	Male ICR mice	Chronic constraint pressure, TST	Saffron extract	28 days	Increase in DA and CREB serum levels	[202]
19	Homology of medicine and food	Schisandra Chinensis	Male Kunming mice	FST	SCE	4 consecutive days	Effects on NE, DA, GABA, and Glu systems	[203]

Note: FST: Forced Swimming Test; CAT: Catalase; SOD: Super Oxide Dismutase; GSH: Glutathione; OVX: Ovariectomized; CSDS: Chronic Social Defeat Stress; TST: Tail Suspension Test; LPS: Lipopolysaccharide; ω-3PUFA: ω-3-Polyunsaturated Fatty Acids; ERK: Extracellular Signal-Regulated Kinase; DA: Dopamine; CUS: Chronic Unpredictable Stress; AECSF: Aqueous Extract of Channa striatus Fillet; FSL: Flinders Sensitive Line; CMS: Chronic Mild Stress; CUMS: Chronic Unpredictable Mild Stress; PSD: Post-Stroke Depression; OFT: Open Field Test; CREB: Cyclic AMP Response Element Binding Protein; SCE: Schisandra Chinensis Extracts; GABA: Gamma-Aminobutyric Acid.

**Table 3:** Possible antidepressant mechanism of active ingredients of food.

NO.	Food Effective Constituent	Molecular Formula	Structural Formula	Source	Putative Antidepressant Mechanisms	References
1	Quercetin	C_15_H_10_O_7_	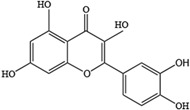	A variety of vegetables and fruits, such as apple peel and citrus fruits	Improvement of HPA axis dysfunction and reduction in inflammation	[204]
2	Eicosapentaenoic (EPA) and docosahexaenoic (DHA)	C_20_H_30_O_2_	EPA	Fish oil	Increase in serotonergic neurotransmission	[205,206,207]
			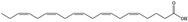			
		C_22_H_32_O_2_	DHA			
			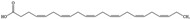			
3	Caffeine	C_8_H_10_N_4_O_2_	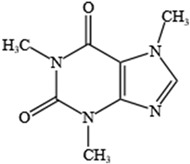	Coffee, tea, chocolate, and caffeinated beverages	Enhancement of extracellular levels of DA and NE	[208,209,210]
4	Vitamin B	C_12_H_17_ClN_4_OS	Vitamin B1 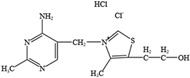	Raw and fermented fish sauce and certain vegetables consumed primarily in Africa and Asia	Regulation of oxidative stress and inflammation	[211,212]
5	Vitamin B	C_17_H_20_N_4_O_6_	Vitamin B2 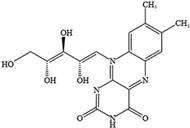	Meat, milk, fish, nuts, eggs, vegetables, and some fruits	Regulation of oxidative stress and inflammation	[213,214]
6	Vitamin C	C_6_H_8_O_6_	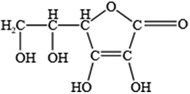	Fruits, vegetables, potatoes, and soft drinks (fruit juices)	Regulation of monoamine and glutamate neurotransmitter systems	[215,216]
7	Vitamin D	Vitamin D2	Vitamin D2	Many products of animal origin, such as deep-sea fish and animal liver	Regulation of monoamine and glutamate neurotransmitter systems	[217,218,219]
		C_28_H_44_O	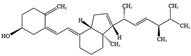			
		Vitamin D3	Vitamin D3			
		C_27_H_44_O	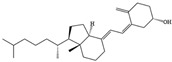			
8	Ginsenoside Rb1	C_54_H_92_O_23_	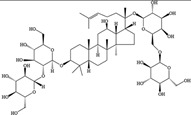	Ginseng	Inhibition of oxidation, apoptosis, inflammation, and autophagy	[220,221,222]
9	Curcumin	C_21_H_20_O_6_	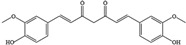	Turmeric plant	Increase in monoamines and BDNF levels and inhibition of pro-inflammatory factors and neuronal apoptosis in the brain	[223,224]
10	Piperine	C_17_H_19_NO_3_	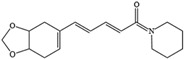	Pepper	Regulation of oxidative stress and inflammation	[225,226]

## Data Availability

Not applicable.

## References

[1] Kruizinga J., Liemburg E., Burger H., Cipriani A., Geddes J., Robertson L., Vogelaar B., Nolen W.A. (2021). Pharmacological treatment for psychotic depression. Cochrane Database Syst. Rev..

[2] van Bronswijk S., Moopen N., Beijers L., Ruhe H.G., Peeters F. (2019). Effectiveness of psychotherapy for treatment-resistant depression: A meta-analysis and meta-regression. Psychol. Med..

[3] Harmer C.J., Duman R.S., Cowen P.J. (2017). How do antidepressants work? New perspectives for refining future treatment approaches. Lancet Psychiatry.

[4] Alexopoulos G.S., Streim J., Carpenter D., Docherty J.P., Expert Consensus Panel for Using Antipsychotic Drugs in Older Patients (2004). Using antipsychotic agents in older patients. J. Clin. Psychiatry.

[5] Khawam E.A., Laurencic G., Malone D.A. (2006). Side effects of antidepressants: An overview. Clevel. Clin. J. Med..

[6] Kris-Etherton P.M., Petersen K.S., Hibbeln J.R., Hurley D., Kolick V., Peoples S., Rodriguez N., Woodward-Lopez G. (2021). Nutrition and behavioral health disorders: Depression and anxiety. Nutr. Rev..

[7] Tolkien K., Bradburn S., Murgatroyd C. (2019). An anti-inflammatory diet as a potential intervention for depressive disorders: A systematic review and meta-analysis. Clin. Nutr..

[8] Manosso L.M., Arent C.O., Borba L.A., Abelaira H.M., Reus G.Z. (2022). Natural Phytochemicals for the Treatment of Major Depressive Disorder: A Mini-Review of Pre- and Clinical Studies. CNS Neurol. Disord. Drug Targets.

[9] Donoso F., Cryan J.F., Olavarria-Ramirez L., Nolan Y.M., Clarke G. (2022). Inflammation, Lifestyle Factors, and the Microbiome-Gut-Brain Axis: Relevance to Depression and Antidepressant Action. Clin. Pharmacol. Ther..

[10] Masand P.S., Gupta S. (1999). Selective serotonin-reuptake inhibitors: An update. Harv. Rev. Psychiatry.

[11] Ho D. (2012). Antidepressants and the FDA’s black-box warning: Determining a rational public policy in the absence of sufficient evidence. AMA J. Ethics.

[12] Lambert O., Bourin M. (2002). SNRIs: Mechanism of action and clinical features. Expert Rev. Neurother..

[13] Sanchez C., Asin K.E., Artigas F. (2015). Vortioxetine, a novel antidepressant with multimodal activity: Review of preclinical and clinical data. Pharmacol. Ther..

[14] Burch R. (2019). Antidepressants for Preventive Treatment of Migraine. Curr. Treat. Options Neurol..

[15] Jaffer K.Y., Chang T., Vanle B., Dang J., Steiner A.J., Loera N., Abdelmesseh M., Danovitch I., Ishak W.W. (2017). Trazodone for Insomnia: A Systematic Review. Innov. Clin. Neurosci..

[16] Davis R., Whittington R., Bryson H.M. (1997). Nefazodone. A review of its pharmacology and clinical efficacy in the management of major depression. Drugs.

[17] Grabowski Ł. (2021). Monoamine oxidase inhibitors (MAOI): Pharmacology, metabolism and application in the treatment of depression. Postep. Biochem..

[18] Hyman Rapaport M. (2007). Translating the evidence on atypical depression into clinical practice. J. Clin. Psychiatry.

[19] Feighner J.P. (1999). Mechanism of action of antidepressant medications. J. Clin. Psychiatry.

[20] Wang S.M., Han C., Bahk W.M., Lee S.J., Patkar A.A., Masand P.S., Pae C.U. (2018). Addressing the Side Effects of Contemporary Antidepressant Drugs: A Comprehensive Review. Chonnam Med. J..

[21] Aringhieri S., Carli M., Kolachalam S., Verdesca V., Cini E., Rossi M., McCormick P.J., Corsini G.U., Maggio R., Scarselli M. (2018). Molecular targets of atypical antipsychotics: From mechanism of action to clinical differences. Pharmacol. Ther..

[22] Anttila S.A., Leinonen E.V. (2001). A review of the pharmacological and clinical profile of mirtazapine. CNS Drug Rev..

[23] Protti M., Mandrioli R., Marasca C., Cavalli A., Serretti A., Mercolini L. (2020). New-generation, non-SSRI antidepressants: Drug-drug interactions and therapeutic drug monitoring. Part 2: NaSSAs, NRIs, SNDRIs, MASSAs, NDRIs, and others. Med. Res. Rev..

[24] Wang P., Jing C., Yu P., Lu M., Xu X., Pei Q., Yan F. (2021). Profiling the structural determinants of aminoketone derivatives as hNET and hDAT reuptake inhibitors by field-based QSAR based on molecular docking. Technol. Health Care Off. J. Eur. Soc. Eng. Med..

[25] Hu L.Y., Liu C.J., Lu T., Hu T.M., Tsai C.F., Hu Y.W., Shen C.C., Chang Y.S., Chen M.H., Teng C.J. (2013). Delayed onset urticaria in depressive patients with bupropion prescription: A nationwide population-based study. PLoS ONE.

[26] Bunney W.E., Davis J.M. (1965). Norepinephrine in depressive reactions. A review. Arch. Gen. Psychiatry.

[27] Kohler S., Cierpinsky K., Kronenberg G., Adli M. (2016). The serotonergic system in the neurobiology of depression: Relevance for novel antidepressants. J. Psychopharmacol..

[28] Blier P. (2016). Neurobiology of depression and mechanism of action of depression treatments. J. Clin. Psychiatry.

[29] Gu S., Wang F., Patel N.P., Bourgeois J.A., Huang J.H. (2019). A Model for Basic Emotions Using Observations of Behavior in Drosophila. Front. Psychol..

[30] Jiang Y., Zou D., Li Y., Gu S., Dong J., Ma X., Xu S., Wang F., Huang J.H. (2022). Monoamine Neurotransmitters Control Basic Emotions and Affect Major Depressive Disorders. Pharmaceuticals.

[31] Zanos P., Moaddel R., Morris P.J., Riggs L.M., Highland J.N., Georgiou P., Pereira E.F.R., Albuquerque E.X., Thomas C.J., Zarate C.A. (2018). Ketamine and Ketamine Metabolite Pharmacology: Insights into Therapeutic Mechanisms. Pharmacol. Rev..

[32] Kucukibrahimoglu E., Saygin M.Z., Caliskan M., Kaplan O.K., Unsal C., Goren M.Z. (2009). The change in plasma GABA, glutamine and glutamate levels in fluoxetine- or S-citalopram-treated female patients with major depression. Eur. J. Clin. Pharmacol..

[33] Lepack A.E., Bang E., Lee B., Dwyer J.M., Duman R.S. (2016). Fast-acting antidepressants rapidly stimulate ERK signaling and BDNF release in primary neuronal cultures. Neuropharmacology.

[34] Aleksandrova L.R., Phillips A.G. (2021). Neuroplasticity as a convergent mechanism of ketamine and classical psychedelics. Trends Pharmacol. Sci..

[35] Fang Y., Ding X., Zhang Y., Cai L., Ge Y., Ma K., Xu R., Li S., Song M., Zhu H. (2022). Fluoxetine inhibited the activation of A1 reactive astrocyte in a mouse model of major depressive disorder through astrocytic 5-HT(2B)R/beta-arrestin2 pathway. J. Neuroinflamm..

[36] Peng C.H., Chiou S.H., Chen S.J., Chou Y.C., Ku H.H., Cheng C.K., Yen C.J., Tsai T.H., Chang Y.L., Kao C.L. (2008). Neuroprotection by Imipramine against lipopolysaccharide-induced apoptosis in hippocampus-derived neural stem cells mediated by activation of BDNF and the MAPK pathway. Eur. Neuropsychopharmacol..

[37] Bjorkholm C., Monteggia L.M. (2016). BDNF—A key transducer of antidepressant effects. Neuropharmacology.

[38] Yirmiya R., Rimmerman N., Reshef R. (2015). Depression as a microglial disease. Trends Neurosci..

[39] Arauchi R., Hashioka S., Tsuchie K., Miyaoka T., Tsumori T., Limoa E., Azis I.A., Oh-Nishi A., Miura S., Otsuki K. (2018). Gunn rats with glial activation in the hippocampus show prolonged immobility time in the forced swimming test and tail suspension test. Brain Behav..

[40] Maes M., Vandoolaeghe E., Ranjan R., Bosmans E., Bergmans R., Desnyder R. (1995). Increased serum interleukin-1-receptor-antagonist concentrations in major depression. J. Affect. Disord..

[41] Halbreich U., Asnis G.M., Shindledecker R., Zumoff B., Nathan R.S. (1985). Cortisol secretion in endogenous depression. I. Basal plasma levels. Arch. Gen. Psychiatry.

[42] Carroll B.J., Feinberg M., Greden J.F., Tarika J., Albala A.A., Haskett R.F., James N.M., Kronfol Z., Lohr N., Steiner M. (1981). A specific laboratory test for the diagnosis of melancholia. Standardization, validation, and clinical utility. Arch. Gen. Psychiatry.

[43] Desbonnet L., Clarke G., Traplin A., O’Sullivan O., Crispie F., Moloney R.D., Cotter P.D., Dinan T.G., Cryan J.F. (2015). Gut microbiota depletion from early adolescence in mice: Implications for brain and behaviour. Brain Behav. Immun..

[44] Frohlich E.E., Farzi A., Mayerhofer R., Reichmann F., Jacan A., Wagner B., Zinser E., Bordag N., Magnes C., Frohlich E. (2016). Cognitive impairment by antibiotic-induced gut dysbiosis: Analysis of gut microbiota-brain communication. Brain Behav. Immun..

[45] Fitzpatrick P.F. (1999). Tetrahydropterin-dependent amino acid hydroxylases. Annu. Rev. Biochem..

[46] Kim D.Y., Camilleri M. (2000). Serotonin: A mediator of the brain-gut connection. Am. J. Gastroenterol..

[47] Jenkins T.A., Nguyen J.C., Polglaze K.E., Bertrand P.P. (2016). Influence of Tryptophan and Serotonin on Mood and Cognition with a Possible Role of the Gut-Brain Axis. Nutrients.

[48] Wang L., Zhu Z., Hou W., Zhang X., He Z., Yuan W., Yang Y., Zhang S., Jia R., Tai F. (2019). Serotonin Signaling Trough Prelimbic 5-HT1A Receptors Modulates CSDS-Induced Behavioral Changes in Adult Female Voles. Int. J. Neuropsychopharmacol..

[49] Goda R., Otsuka T., Iwamoto A., Kawai M., Shibata S., Furuse M., Yasuo S. (2015). Serotonin levels in the dorsal raphe nuclei of both chipmunks and mice are enhanced by long photoperiod, but brain dopamine level response to photoperiod is species-specific. Neurosci. Lett..

[50] Whitney M.S., Shemery A.M., Yaw A.M., Donovan L.J., Glass J.D., Deneris E.S. (2016). Adult Brain Serotonin Deficiency Causes Hyperactivity, Circadian Disruption, and Elimination of Siestas. J. Neurosci..

[51] David D.J., Gardier A.M. (2016). The pharmacological basis of the serotonin system: Application to antidepressant response. Encephale.

[52] Cryan J.F., Leonard B.E. (2000). 5-HT_1A_ and beyond: The role of serotonin and its receptors in depression and the antidepressant response. Hum. Psychopharmacol..

[53] Detke M.J., Wieland S., Lucki I. (1995). Blockade of the antidepressant-like effects of 8-OH-DPAT, buspirone and desipramine in the rat forced swim test by 5HT_1A_ receptor antagonists. Psychopharmacology.

[54] Garcia-Garcia A.L., Newman-Tancredi A., Leonardo E.D. (2014). P5-HT_1A_ receptors in mood and anxiety: Recent insights into autoreceptor versus heteroreceptor function. Psychopharmacology.

[55] Cheetham S.C., Crompton M.R., Katona C.L., Horton R.W. (1990). Brain 5-HT_1_ binding sites in depressed suicides. Psychopharmacology.

[56] Kim M.H., Leem Y.H. (2014). Chronic exercise improves repeated restraint stress-induced anxiety and depression through 5HT1A receptor and cAMP signaling in hippocampus. J. Exerc. Nutr. Biochem..

[57] Kennett G.A., Dourish C.T., Curzon G. (1987). Antidepressant-like action of 5-HT1A agonists and conventional antidepressants in an animal model of depression. Eur. J. Pharmacol..

[58] Wang L., Zhang Y., Du X., Ding T., Gong W., Liu F. (2019). Review of antidepressants in clinic and active ingredients of traditional Chinese medicine targeting 5-HT1A receptors. Biomed. Pharmacother..

[59] Jones M.D., Lucki I. (2005). Sex differences in the regulation of serotonergic transmission and behavior in 5-HT receptor knockout mice. Neuropsychopharmacology.

[60] Tatarczynska E., Klodzinska A., Stachowicz K., Chojnacka-Wojcik E. (2004). Effects of a selective 5-HT_1B_ receptor agonist and antagonists in animal models of anxiety and depression. Behav. Pharmacol..

[61] Hasegawa S., Nishi K., Watanabe A., Overstreet D.H., Diksic M. (2006). Brain 5-HT synthesis in the Flinders Sensitive Line rat model of depression: An autoradiographic study. Neurochem. Int..

[62] Sun X., Zhang T., Zhao Y., Cai E., Zhu H., Liu S. (2020). Panaxynol attenuates CUMS-induced anxiety and depressive-like behaviors via regulating neurotransmitters, synapses and the HPA axis in mice. Food Funct..

[63] Aznar S., Hervig M.E.-S. (2016). The 5-HT2A serotonin receptor in executive function: Implications for neuropsychiatric and neurodegenerative diseases. Neurosci. Biobehav. Rev..

[64] Tsujita N., Akamatsu Y., Nishida M.M., Hayashi T., Moritani T. (2019). Effect of Tryptophan, Vitamin B_6_, and Nicotinamide-Containing Supplement Loading between Meals on Mood and Autonomic Nervous System Activity in Young Adults with Subclinical Depression: A Randomized, Double-Blind, and Placebo-Controlled Study. J. Nutr. Sci. Vitaminol..

[65] Tanke M.A., Alserda E., Doornbos B., van der Most P.J., Goeman K., Postema F., Korf J. (2008). Low tryptophan diet increases stress-sensitivity, but does not affect habituation in rats. Neurochem. Int..

[66] Mayerhofer A., Smith G.D., Danilchik M., Levine J.E., Wolf D.P., Dissen G.A., Ojeda S.R. (1998). Oocytes are a source of catecholamines in the primate ovary: Evidence for a cell-cell regulatory loop. Proc. Natl. Acad. Sci. USA.

[67] Nagatsu T., Levitt M., Udenfriend S. (1964). Tyrosine Hydroxylase: The Initial Step in Norepinephrine Biosynthesis. J. Biol. Chem..

[68] Martin-Hernandez D., Pereira M.P., Tendilla-Beltran H., Madrigal J.L.M., Garcia-Bueno B., Leza J.C., Caso J.R. (2019). Modulation of Monoaminergic Systems by Antidepressants in the Frontal Cortex of Rats after Chronic Mild Stress Exposure. Mol. Neurobiol..

[69] Kremer M., Salvat E., Muller A., Yalcin I., Barrot M. (2016). Antidepressants and gabapentinoids in neuropathic pain: Mechanistic insights. Neuroscience.

[70] Brunello N., Mendlewicz J., Kasper S., Leonard B., Montgomery S., Nelson J.C., Paykel E., Versiani M., Racagni G. (2002). The role of noradrenaline and selective noradrenaline reuptake inhibition in depression. Eur. Neuropsychopharmacol..

[71] Li X.R., Sun N., Xu Y., Wang Y.F., Li S.P., Du Q.R., Peng J.Y., Luo J.X., Zhang K.R. (2012). The norepinephrine transporter gene is associated with the retardation symptoms of major depressive disorder in the Han Chinese population. Neural Regen. Res..

[72] Liu D., Hu X.Y., Xia H.J., Wang L.J., Shi P., Chen X.P., Zhou Q.X. (2019). Antidepressant effect of venlafaxine in chronic unpredictable stress: Evidence of the involvement of key enzymes responsible for monoamine neurotransmitter synthesis and metabolism. Mol. Med. Rep..

[73] Alfinito P.D., Huselton C., Chen X., Deecher D.C. (2006). Pharmacokinetic and pharmacodynamic profiles of the novel serotonin and norepinephrine reuptake inhibitor desvenlafaxine succinate in ovariectomized Sprague-Dawley rats. Brain Res..

[74] Liebe T., Li M., Colic L., Munk M.H.J., Sweeney-Reed C.M., Woelfer M., Kretzschmar M.A., Steiner J., von During F., Behnisch G. (2018). Ketamine influences the locus coeruleus norepinephrine network, with a dependency on norepinephrine transporter genotype—A placebo controlled fMRI study. NeuroImage Clin..

[75] Bui B.V., Fortune B. (2004). Ganglion cell contributions to the rat full-field electroretinogram. J. Physiol..

[76] Iro C.M., Hamati R., El Mansari M., Blier P. (2021). Repeated but Not Single Administration of Ketamine Prolongs Increases of the Firing Activity of Norepinephrine and Dopamine Neurons. Int. J. Neuropsychopharmacol..

[77] Franco R., Reyes-Resina I., Navarro G. (2021). Dopamine in Health and Disease: Much More Than a Neurotransmitter. Biomedicines.

[78] Hoefig C.S., Renko K., Piehl S., Scanlan T.S., Bertoldi M., Opladen T., Hoffmann G.F., Klein J., Blankenstein O., Schweizer U. (2012). Does the aromatic L-amino acid decarboxylase contribute to thyronamine biosynthesis?. Mol. Cell Endocrinol..

[79] Pendleton R.G., Rasheed A., Paluru P., Joyner J., Jerome N., Meyers R.D., Hillman R. (2005). A developmental role for catecholamines in Drosophila behavior. Pharmacol. Biochem. Behav..

[80] Ishikawa T., Okano M., Minami A., Tsunekawa H., Satoyoshi H., Tsukamoto Y., Takahata K., Muraoka S. (2019). Selegiline ameliorates depression-like behaviors in rodents and modulates hippocampal dopaminergic transmission and synaptic plasticity. Behav. Brain Res..

[81] Camardese G., Di Giuda D., Di Nicola M., Cocciolillo F., Giordano A., Janiri L., Guglielmo R. (2014). Imaging studies on dopamine transporter and depression: A review of literature and suggestions for future research. J. Psychiatr. Res..

[82] Nikolaus S., Mamlins E., Hautzel H., Muller H.W. (2019). Acute anxiety disorder, major depressive disorder, bipolar disorder and schizophrenia are related to different patterns of nigrostriatal and mesolimbic dopamine dysfunction. Rev. Neurosci..

[83] Shen M.Y., Perreault M.L., Bambico F.R., Jones-Tabah J., Cheung M., Fan T., Nobrega J.N., George S.R. (2015). Rapid anti-depressant and anxiolytic actions following dopamine D1–D2 receptor heteromer inactivation. Eur. Neuropsychopharmacol..

[84] Hasbi A., Nguyen T., Rahal H., Manduca J.D., Miksys S., Tyndale R.F., Madras B.K., Perreault M.L., George S.R. (2020). Sex difference in dopamine D1–D2 receptor complex expression and signaling affects depression- and anxiety-like behaviors. Biol. Sex Differ..

[85] Desormeaux C., Demars F., Davenas E., Jay T.M., Lavergne F. (2020). Selective activation of D1 dopamine receptors exerts antidepressant-like activity in rats. J. Psychopharmacol..

[86] Rocchetti J., Isingrini E., Dal Bo G., Sagheby S., Menegaux A., Tronche F., Levesque D., Moquin L., Gratton A., Wong T.P. (2015). Presynaptic D2 dopamine receptors control long-term depression expression and memory processes in the temporal hippocampus. Biol. Psychiatry.

[87] Moraga-Amaro R., Gonzalez H., Pacheco R., Stehberg J. (2014). Dopamine receptor D3 deficiency results in chronic depression and anxiety. Behav. Brain Res..

[88] Perona M.T., Waters S., Hall F.S., Sora I., Lesch K.P., Murphy D.L., Caron M., Uhl G.R. (2008). Animal models of depression in dopamine, serotonin, and norepinephrine transporter knockout mice: Prominent effects of dopamine transporter deletions. Behav. Pharmacol..

[89] Romeo B., Blecha L., Locatelli K., Benyamina A., Martelli C. (2018). Meta-analysis and review of dopamine agonists in acute episodes of mood disorder: Efficacy and safety. J. Psychopharmacol..

[90] Conway C.R., Chibnall J.T., Cumming P., Mintun M.A., Gebara M.A., Perantie D.C., Price J.L., Cornell M.E., McConathy J.E., Gangwani S. (2014). Antidepressant response to aripiprazole augmentation associated with enhanced FDOPA utilization in striatum: A preliminary PET study. Psychiatry Res..

[91] Moriguchi S., Takamiya A., Noda Y., Horita N., Wada M., Tsugawa S., Plitman E., Sano Y., Tarumi R., ElSalhy M. (2019). Glutamatergic neurometabolite levels in major depressive disorder: A systematic review and meta-analysis of proton magnetic resonance spectroscopy studies. Mol. Psychiatry.

[92] Fukumoto K., Iijima M., Funakoshi T., Chaki S. (2018). 5-HT_1A_ receptor stimulation in the medial prefrontal cortex mediates the antidepressant effects of mGlu2/3 receptor antagonist in mice. Neuropharmacology.

[93] Palucha-Poniewiera A., Branski P., Wieronska J.M., Stachowicz K., Slawinska A., Pilc A. (2014). The antidepressant-like action of mGlu5 receptor antagonist, MTEP, in the tail suspension test in mice is serotonin dependent. Psychopharmacology.

[94] Podkowa K., Pilc A., Podkowa A., Sałat K., Marciniak M., Pałucha-Poniewiera A. (2018). The potential antidepressant action and adverse effects profile of scopolamine co-administered with the mGlu7 receptor allosteric agonist AMN082 in mice. Neuropharmacology.

[95] Chen Y.P., Wang C., Xu J.P. (2019). Chronic unpredictable mild stress induced depression-like behaviours and glutamate-glutamine cycling dysfunctions in both blood and brain of mice. Pharm. Biol..

[96] de Lima D.S., Francisco E.D., Lima C.B., Guedes R.C. (2017). Neonatal L-glutamine modulates anxiety-like behavior, cortical spreading depression, and microglial immunoreactivity: Analysis in developing rats suckled on normal size- and large size litters. Amino Acids.

[97] Greger I.H., Watson J.F., Cull-Candy S.G. (2017). Structural and Functional Architecture of AMPA-Type Glutamate Receptors and Their Auxiliary Proteins. Neuron.

[98] Gerhard D.M., Pothula S., Liu R.J., Wu M., Li X.Y., Girgenti M.J., Taylor S.R., Duman C.H., Delpire E., Picciotto M. (2020). GABA interneurons are the cellular trigger for ketamine’s rapid antidepressant actions. J. Clin. Investig..

[99] Kantrowitz J.T., Dong Z., Milak M.S., Rashid R., Kegeles L.S., Javitt D.C., Lieberman J.A., John Mann J. (2021). Ventromedial prefrontal cortex/anterior cingulate cortex Glx, glutamate, and GABA levels in medication-free major depressive disorder. Transl. Psychiatry.

[100] Milak M.S., Proper C.J., Mulhern S.T., Parter A.L., Kegeles L.S., Ogden R.T., Mao X., Rodriguez C.I., Oquendo M.A., Suckow R.F. (2016). A pilot in vivo proton magnetic resonance spectroscopy study of amino acid neurotransmitter response to ketamine treatment of major depressive disorder. Mol. Psychiatry.

[101] Petroff O.A. (2002). GABA and glutamate in the human brain. Neuroscientist.

[102] Ma K., Zhang H., Wang S., Wang H., Wang Y., Liu J., Song X., Dong Z., Han X., Zhang Y. (2019). The molecular mechanism underlying GABAergic dysfunction in nucleus accumbens of depression-like behaviours in mice. J. Cell Mol. Med..

[103] Sanacora G., Gueorguieva R., Epperson C.N., Wu Y.T., Appel M., Rothman D.L., Krystal J.H., Mason G.F. (2004). Subtype-specific alterations of gamma-aminobutyric acid and glutamate in patients with major depression. Arch. Gen. Psychiatry.

[104] Liang M., Zhong H.Q., Rong J., Li Y.C., Zhu C.T., Zhou L., Zhou R. (2019). Postnatal Lipopolysaccharide Exposure Impairs Adult Neurogenesis and Causes Depression-like Behaviors through Astrocytes Activation Triggering GABAA Receptor Downregulation. Neuroscience.

[105] Dafre A.L., Rosa J.M., Rodrigues A.L.S., Cunha M.P. (2020). Multiple cellular targets involved in the antidepressant-like effect of glutathione. Chem. Biol. Interact..

[106] Edinoff A.N., Odisho A.S., Lewis K., Kaskas A., Hunt G., Cornett E.M., Kaye A.D., Kaye A., Morgan J., Barrilleaux P.S. (2021). Brexanolone, a GABA(A) Modulator, in the Treatment of Postpartum Depression in Adults: A Comprehensive Review. Front. Psychiatry.

[107] Chuang C.Y., Shi Y.C., You H.P., Lo Y.H., Pan T.M. (2011). Antidepressant effect of GABA-rich monascus-fermented product on forced swimming rat model. J. Agric. Food Chem..

[108] Simpson S.M., Hickey A.J., Baker G.B., Reynolds J.N., Beninger R.J. (2012). The antidepressant phenelzine enhances memory in the double Y-maze and increases GABA levels in the hippocampus and frontal cortex of rats. Pharmacol. Biochem. Behav..

[109] Esvald E.E., Tuvikene J., Sirp A., Patil S., Bramham C.R., Timmusk T. (2020). CREB Family Transcription Factors Are Major Mediators of BDNF Transcriptional Autoregulation in Cortical Neurons. J. Neurosci..

[110] Taliaz D., Stall N., Dar D.E., Zangen A. (2010). Knockdown of brain-derived neurotrophic factor in specific brain sites precipitates behaviors associated with depression and reduces neurogenesis. Mol. Psychiatry.

[111] Nibuya M., Morinobu S., Duman R.S. (1995). Regulation of BDNF and trkB mRNA in rat brain by chronic electroconvulsive seizure and antidepressant drug treatments. J. Neurosci. Off. J. Soc. Neurosci..

[112] Duman R.S., Monteggia L.M. (2006). A neurotrophic model for stress-related mood disorders. Biol. Psychiatry.

[113] Fan J.F., Tang Z.H., Wang S.Y., Lei S., Zhang B., Tian S.W. (2021). Ketamine enhances novel object recognition memory reconsolidation via the BDNF/TrkB pathway in mice. Physiol. Behav..

[114] Chen B., Dowlatshahi D., MacQueen G.M., Wang J.F., Young L.T. (2001). Increased hippocampal BDNF immunoreactivity in subjects treated with antidepressant medication. Biol. Psychiatry.

[115] Calabrese F., Molteni R., Cattaneo A., Macchi F., Racagni G., Gennarelli M., Ellenbroek B.A., Riva M.A. (2010). Long-Term duloxetine treatment normalizes altered brain-derived neurotrophic factor expression in serotonin transporter knockout rats through the modulation of specific neurotrophin isoforms. Mol. Pharmacol..

[116] Wook Koo J., Labonte B., Engmann O., Calipari E.S., Juarez B., Lorsch Z., Walsh J.J., Friedman A.K., Yorgason J.T., Han M.H. (2016). Essential Role of Mesolimbic Brain-Derived Neurotrophic Factor in Chronic Social Stress-Induced Depressive Behaviors. Biol. Psychiatry.

[117] Taliaz D., Nagaraj V., Haramati S., Chen A., Zangen A. (2013). Altered brain-derived neurotrophic factor expression in the ventral tegmental area, but not in the hippocampus, is essential for antidepressant-like effects of electroconvulsive therapy. Biol. Psychiatry.

[118] Lu C.L., Ren J., Mo J.W., Fan J., Guo F., Chen L.Y., Wen Y.L., Li S.J., Fang Y.Y., Wu Z.F. (2022). Glucocorticoid Receptor-Dependent Astrocytes Mediate Stress Vulnerability. Biol. Psychiatry.

[119] Bender C.L., Sun X., Farooq M., Yang Q., Davison C., Maroteaux M., Huang Y.S., Ishikawa Y., Liu S.J. (2020). Emotional Stress Induces Structural Plasticity in Bergmann Glial Cells via an AC5-CPEB3-GluA1 Pathway. J. Neurosci..

[120] Liu Q.O., Li B., Zhu H.Y., Wang Y.Q., Yu J., Wu G.C. (2011). Glia atrophy in the hippocampus of chronic unpredictable stress-induced depression model rats is reversed by electroacupuncture treatment. J. Affect. Disorders..

[121] Wang Q., Kong Y., Lin S., Wu D.Y., Hu J., Huang L., Zang W.S., Li X.W., Yang J.M., Gao T.M. (2021). The ATP Level in the mPFC Mediates the Antidepressant Effect of Calorie Restriction. Neurosci. Bull..

[122] Li Y., Luo Y., Tang J., Liang X., Wang J., Xiao Q., Zhu P., Xiao K., Jiang L., Dou X. (2021). The positive effects of running exercise on hippocampal astrocytes in a rat model of depression. Transl. Psychiatry.

[123] Luo Y., Xiao Q., Wang J., Jiang L., Hu M., Jiang Y., Tang J., Liang X., Qi Y., Dou X. (2019). Running exercise protects oligodendrocytes in the medial prefrontal cortex in chronic unpredictable stress rat model. Transl. Psychiatry.

[124] Tang J., Liang X., Zhang Y., Chen L., Wang F., Tan C., Luo Y., Xiao Q., Chao F., Zhang L. (2019). The effects of running exercise on oligodendrocytes in the hippocampus of rats with depression induced by chronic unpredictable stress. Brain Res. Bull..

[125] Dionisie V., Ciobanu A.M., Toma V.A., Manea M.C., Baldea I., Olteanu D., Sevastre-Berghian A., Clichici S., Manea M., Riga S. (2021). Escitalopram Targets Oxidative Stress, Caspase-3, BDNF and MeCP2 in the Hippocampus and Frontal Cortex of a Rat Model of Depression Induced by Chronic Unpredictable Mild Stress. Int. J. Mol. Sci..

[126] Lago N., Kaufmann F.N., Negro-Demontel M.L., Ali-Ruiz D., Ghisleni G., Rego N., Arcas-Garcia A., Vitureira N., Jansen K., Souza L.M. (2020). CD300f immunoreceptor is associated with major depressive disorder and decreased microglial metabolic fitness. Proc. Natl. Acad. Sci. USA.

[127] Kwon S.H., Han J.K., Choi M., Kwon Y.J., Kim S.J., Yi E.H., Shin J.C., Cho I.H., Kim B.H., Jeong Kim S. (2017). Dysfunction of Microglial STAT3 Alleviates Depressive Behavior via Neuron-Microglia Interactions. Neuropsychopharmacology.

[128] Zhu H.X., Cheng L.J., Ou Yang R.W., Li Y.Y., Liu J., Dai D., Wang W., Yang N., Li Y. (2020). Reduced Amygdala Microglial Expression of Brain-Derived Neurotrophic Factor and Tyrosine Kinase Receptor B (TrkB) in a Rat Model of Poststroke Depression. Med. Sci. Monit..

[129] Yue N., Huang H., Zhu X., Han Q., Wang Y., Li B., Liu Q., Wu G., Zhang Y., Yu J. (2017). Activation of P2X7 receptor and NLRP3 inflammasome assembly in hippocampal glial cells mediates chronic stress-induced depressive-like behaviors. J. Neuroinflamm..

[130] Howren M.B., Lamkin D.M., Suls J. (2009). Associations of depression with C-reactive protein, IL-1, and IL-6: A meta-analysis. Psychosom. Med..

[131] Lindqvist D., Dhabhar F.S., James S.J., Hough C.M., Jain F.A., Bersani F.S., Reus V.I., Verhoeven J.E., Epel E.S., Mahan L. (2017). Oxidative stress, inflammation and treatment response in major depression. Psychoneuroendocrinology.

[132] Li W., Ali T., He K., Liu Z., Shah F.A., Ren Q., Liu Y., Jiang A., Li S. (2021). Ibrutinib alleviates LPS-induced neuroinflammation and synaptic defects in a mouse model of depression. Brain Behav. Immun..

[133] Zhao X., Cao F., Liu Q., Li X., Xu G., Liu G., Zhang Y., Yang X., Yi S., Xu F. (2019). Behavioral, inflammatory and neurochemical disturbances in LPS and UCMS-induced mouse models of depression. Behav. Brain Res..

[134] Zhao Y., Sun X., Zhang T., Liu S., Cai E., Zhu H. (2021). Study on the antidepressant effect of panaxynol through the IkappaB-alpha/NF-kappaB signaling pathway to inhibit the excessive activation of BV-2 microglia. Biomed. Pharmacother..

[135] Beurel E., Toups M., Nemeroff C.B. (2020). The Bidirectional Relationship of Depression and Inflammation: Double Trouble. Neuron.

[136] Wang Y., Wang S., Xin Y., Zhang J., Wang S., Yang Z., Liu C. (2021). Hydrogen sulfide alleviates the anxiety-like and depressive-like behaviors of type 1 diabetic mice via inhibiting inflammation and ferroptosis. Life Sci..

[137] Ilieva K., Tchekalarova J., Atanasova D., Kortenska L., Atanasova M. (2019). Antidepressant agomelatine attenuates behavioral deficits and concomitant pathology observed in streptozotocin-induced model of Alzheimer’s disease in male rats. Horm. Behav..

[138] Rebai R., Jasmin L., Boudah A. (2021). Agomelatine effects on fat-enriched diet induced neuroinflammation and depression-like behavior in rats. Biomed. Pharmacother..

[139] Demuyser T., Bentea E., Deneyer L., Albertini G., Massie A., Smolders I. (2016). Disruption of the HPA-axis through corticosterone-release pellets induces robust depressive-like behavior and reduced BDNF levels in mice. Neurosci. Lett..

[140] Camargo A., Dalmagro A.P., Rikel L., da Silva E.B., Simao da Silva K.A.B., Zeni A.L.B. (2018). Cholecalciferol counteracts depressive-like behavior and oxidative stress induced by repeated corticosterone treatment in mice. Eur. J. Pharmacol..

[141] Mukherjee K., Knisely A., Jacobson L. (2004). Partial glucocorticoid agonist-like effects of imipramine on hypothalamic-pituitary-adrenocortical activity, thymus weight, and hippocampal glucocorticoid receptors in male C57BL/6 mice. Endocrinology.

[142] Liu Z., Zou Y., He M., Yang P., Qu X., Xu L. (2022). Hydroxysafflor yellow A can improve depressive behavior by inhibiting hippocampal inflammation and oxidative stress through regulating HPA axis. J. Biosci..

[143] Schule C. (2007). Neuroendocrinological mechanisms of actions of antidepressant drugs. J. Neuroendocrinol..

[144] Neufeld K.M., Kang N., Bienenstock J., Foster J.A. (2011). Reduced anxiety-like behavior and central neurochemical change in germ-free mice. Neurogastroenterol. Motil..

[145] Huang F., Liu X., Xu S., Hu S., Wang S., Shi D., Wang K., Wang Z., Lin Q., Li S. (2021). Prevotella histicola Mitigated Estrogen Deficiency-Induced Depression via Gut Microbiota-Dependent Modulation of Inflammation in Ovariectomized Mice. Front. Nutr..

[146] Mayer E.A., Tillisch K., Gupta A. (2015). Gut/brain axis and the microbiota. J. Clin. Investig..

[147] Kaluzna-Czaplinska J., Gatarek P., Chirumbolo S., Chartrand M.S., Bjorklund G. (2019). How important is tryptophan in human health?. Crit. Rev. Food Sci. Nutr..

[148] Gao K., Mu C.L., Farzi A., Zhu W.Y. (2020). Tryptophan Metabolism: A Link between the Gut Microbiota and Brain. Adv. Nutr..

[149] Jin M., Qian Z., Yin J., Xu W., Zhou X. (2019). The role of intestinal microbiota in cardiovascular disease. J. Cell Mol. Med..

[150] Jia W., Zhen J., Liu A., Yuan J., Wu X., Zhao P., Zhao L., Li X., Liu Q., Huang G. (2020). Long-Term Vegan Meditation Improved Human Gut Microbiota. Evid. Based Complement. Altern. Med..

[151] Deng Y., Zhou M., Wang J., Yao J., Yu J., Liu W., Wu L., Wang J., Gao R. (2021). Involvement of the microbiota-gut-brain axis in chronic restraint stress: Disturbances of the kynurenine metabolic pathway in both the gut and brain. Gut Microbes.

[152] Xu Q., Jiang M., Gu S., Zhang X., Feng G., Ma X., Xu S., Wu E., Huang J.H., Wang F. (2022). Metabolomics changes in brain-gut axis after unpredictable chronic mild stress. Psychopharmacology.

[153] Kim Y.K., Shin C. (2018). The Microbiota-Gut-Brain Axis in Neuropsychiatric Disorders: Pathophysiological Mechanisms and Novel Treatments. Curr. Neuropharmacol..

[154] Torres J., Mehandru S., Colombel J.F., Peyrin-Biroulet L. (2017). Crohn’s disease. Lancet.

[155] Giuffrè M., Gazzin S., Zoratti C., Llido J.P., Lanza G., Tiribelli C., Moretti R. (2022). Celiac Disease and Neurological Manifestations: From Gluten to Neuroinflammation. Int. J. Mol. Sci..

[156] Veauthier B., Hornecker J.R. (2018). Crohn’s Disease: Diagnosis and Management. Am. Fam. Physician.

[157] Sharma N., Singh K., Senapati S. (2021). Celiac disease poses significant risk in developing depression, anxiety, headache, epilepsy, panic disorder, dysthymia: A meta-analysis. Indian J. Gastroenterol..

[158] Barberio B., Zamani M., Black C.J., Savarino E.V., Ford A.C. (2021). Prevalence of symptoms of anxiety and depression in patients with inflammatory bowel disease: A systematic review and meta-analysis. Lancet Gastroenterol. Hepatol..

[159] Hargreaves D., Mates E., Menon P., Alderman H., Devakumar D., Fawzi W., Greenfield G., Hammoudeh W., He S., Lahiri A. (2022). Strategies and interventions for healthy adolescent growth, nutrition, and development. Lancet.

[160] den Besten G., van Eunen K., Groen A.K., Venema K., Reijngoud D.J., Bakker B.M. (2013). The role of short-chain fatty acids in the interplay between diet, gut microbiota, and host energy metabolism. J. Lipid Res..

[161] Albenberg L.G., Wu G.D. (2014). Diet and the intestinal microbiome: Associations, functions, and implications for health and disease. Gastroenterology.

[162] Peng X.P., Nie C., Guan W.Y., Qiao L.D., Lu L., Cao S.J. (2020). Regulation of Probiotics on Metabolism of Dietary Protein in Intestine. Curr. Protein Pept. Sci..

[163] Zhang K., Wang N., Lu L., Ma X. (2020). Fermentation and Metabolism of Dietary Protein by Intestinal Microorganisms. Curr. Protein Pept. Sci..

[164] Coda R., Rizzello C.G., Pinto D., Gobbetti M. (2012). Selected lactic acid bacteria synthesize antioxidant peptides during sourdough fermentation of cereal flours. Appl. Environ. Microbiol..

[165] Koh A., De Vadder F., Kovatcheva-Datchary P., Backhed F. (2016). From Dietary Fiber to Host Physiology: Short-Chain Fatty Acids as Key Bacterial Metabolites. Cell.

[166] Yang F.Y., Saqib H.S.A., Chen J.H., Ruan Q.Q., Vasseur L., He W.Y., You M.S. (2020). Differential Profiles of Gut Microbiota and Metabolites Associated with Host Shift of Plutella xylostella. Int. J. Mol. Sci..

[167] Shabbir M.A., Mehak F., Khan Z.M., Ahmed W., Haq S., Khan M.R., Bhat Z.F., Aadil R.M. (2022). Delving the role of nutritional psychiatry to mitigate the COVID-19 pandemic induced stress, anxiety and depression. Trends Food Sci. Technol..

[168] Haridas B., Kossoff E.H. (2022). Dietary Treatments for Epilepsy. Neurol. Clin..

[169] Widmer R.J., Flammer A.J., Lerman L.O., Lerman A. (2015). The Mediterranean diet, its components, and cardiovascular disease. Am. J. Med..

[170] Kramer H. (2019). Diet and Chronic Kidney Disease. Adv. Nutr..

[171] Paglia L. (2018). WHO: Healthy diet to prevent chronic diseases and caries. Eur. J. Paediatr. Dent..

[172] Gazerani P. (2020). Migraine and Diet. Nutrients.

[173] Opie R.S., Itsiopoulos C., Parletta N., Sanchez-Villegas A., Akbaraly T.N., Ruusunen A., Jacka F.N. (2017). Dietary recommendations for the prevention of depression. Nutr. Neurosci..

[174] Chatterton M.L., Mihalopoulos C., O’Neil A., Itsiopoulos C., Opie R., Castle D., Dash S., Brazionis L., Berk M., Jacka F. (2018). Economic evaluation of a dietary intervention for adults with major depression (the “SMILES” trial). BMC Public Health.

[175] Stevenson R.J. (2017). Psychological correlates of habitual diet in healthy adults. Psychol. Bull..

[176] Olivan-Blazquez B., Montero-Marin J., Garcia-Toro M., Vicens-Pons E., Serrano-Ripoll M.J., Castro-Gracia A., Sarasa-Bosque M.C., Mendive-Arbeloa J.M., Lopez-Del-Hoyo Y., Garcia-Campayo J. (2018). Facilitators and barriers to modifying dietary and hygiene behaviours as adjuvant treatment in patients with depression in primary care: A qualitative study. BMC Psychiatry.

[177] Konttinen H., Silventoinen K., Sarlio-Lahteenkorva S., Mannisto S., Haukkala A. (2010). Emotional eating and physical activity self-efficacy as pathways in the association between depressive symptoms and adiposity indicators. Am. J. Clin. Nutr..

[178] Cryan J.F., O’Mahony S.M. (2011). The microbiome-gut-brain axis: From bowel to behavior. Neurogastroenterol. Motil..

[179] Carlessi A.S., Borba L.A., Zugno A.I., Quevedo J., Réus G.Z. (2021). Gut microbiota-brain axis in depression: The role of neuroinflammation. Eur. J. Neurosci..

[180] Rutsch A., Kantsjö J.B., Ronchi F. (2020). The Gut-Brain Axis: How Microbiota and Host Inflammasome Influence Brain Physiology and Pathology. Front. Immunol..

[181] Hu B., Das P., Lv X., Shi M., Aa J., Wang K., Duan L., Gilbert J.A., Nie Y., Wu X.L. (2022). Effects of ‘Healthy’ Fecal Microbiota Transplantation against the Deterioration of Depression in Fawn-Hooded Rats. mSystems.

[182] Kronsten V.T., Tranah T.H., Pariante C., Shawcross D.L. (2022). Gut-derived systemic inflammation as a driver of depression in chronic liver disease. J. Hepatol..

[183] Sharon G., Sampson T.R., Geschwind D.H., Mazmanian S.K. (2016). The Central Nervous System and the Gut Microbiome. Cell.

[184] Samad N., Muneer A., Ullah N., Zaman A., Ayaz M.M., Ahmad I. (2017). Banana fruit pulp and peel involved in antianxiety and antidepressant effects while invigorate memory performance in male mice: Possible role of potential antioxidants. Pak. J. Pharm. Sci..

[185] Valdes-Sustaita B., Lopez-Rubalcava C., Gonzalez-Trujano M.E., Garcia-Viguera C., Estrada-Camarena E. (2017). Aqueous Extract of Pomegranate Alone or in Combination with Citalopram Produces Antidepressant-Like Effects in an Animal Model of Menopause: Participation of Estrogen Receptors. Int. J. Mol. Sci..

[186] Zhang J., Ning L., Wang J. (2020). Dietary quercetin attenuates depressive-like behaviors by inhibiting astrocyte reactivation in response to stress. Biochem. Biophys. Res. Commun..

[187] Hu P., Ma L., Wang Y.G., Ye F., Wang C., Zhou W.H., Zhao X. (2017). Genistein, a dietary soy isoflavone, exerts antidepressant-like effects in mice: Involvement of serotonergic system. Neurochem. Int..

[188] Dang R., Zhou X., Tang M., Xu P., Gong X., Liu Y., Jiao H., Jiang P. (2018). Fish oil supplementation attenuates neuroinflammation and alleviates depressive-like behavior in rats submitted to repeated lipopolysaccharide. Eur. J. Nutr..

[189] Zemdegs J., Rainer Q., Grossmann C.P., Rousseau-Ralliard D., Grynberg A., Ribeiro E., Guiard B.P. (2018). Anxiolytic- and Antidepressant-Like Effects of Fish Oil-Enriched Diet in Brain-Derived Neurotrophic Factor Deficient Mice. Front. Neurosci..

[190] Saleem A.M., Taufik Hidayat M., Jais A.M., Fakurazi S., Moklas M.A., Sulaiman M.R., Amom Z., Basir R. (2013). Involvement of monoaminergic system in the antidepressant-like effect of aqueous extract of Channa striatus in mice. Eur. Rev. Med. Pharmacol. Sci..

[191] Pechlivanova D.M., Tchekalarova J.D., Alova L.H., Petkov V.V., Nikolov R.P., Yakimova K.S. (2012). Effect of long-term caffeine administration on depressive-like behavior in rats exposed to chronic unpredictable stress. Behav. Pharmacol..

[192] Tillmann S., Wegener G. (2019). Probiotics reduce risk-taking behavior in the Elevated Plus Maze in the Flinders Sensitive Line rat model of depression. Behav. Brain Res..

[193] Trautmann C., Bock A., Urbach A., Hubner C.A., Engmann O. (2020). Acute vitamin B12 supplementation evokes antidepressant response and alters Ntrk-2. Neuropharmacology.

[194] Zhou Y., Cong Y., Liu H. (2020). Folic acid ameliorates depression-like behaviour in a rat model of chronic unpredictable mild stress. BMC Neurosci..

[195] Moretti M., Budni J., Ribeiro C.M., Rodrigues A.L. (2012). Involvement of different types of potassium channels in the antidepressant-like effect of ascorbic acid in the mouse tail suspension test. Eur. J. Pharmacol..

[196] Xu Y.X., Liang L.Y. (2021). Vitamin D3/vitamin D receptor signaling mitigates symptoms of post-stroke depression in mice by upregulating hippocampal BDNF expression. Neurosci. Res..

[197] Koshkina A., Dudnichenko T., Baranenko D., Fedotova J., Drago F. (2019). Effects of Vitamin D_3_ in Long-Term Ovariectomized Rats Subjected to Chronic Unpredictable Mild Stress: BDNF, NT-3, and NT-4 Implications. Nutrients.

[198] Wang G.L., He Z.M., Zhu H.Y., Gao Y.G., Zhao Y., Yang H., Zhang L.X. (2017). Involvement of serotonergic, noradrenergic and dopaminergic systems in the antidepressant-like effect of ginsenoside Rb1, a major active ingredient of Panax ginseng C.A. Meyer. J. Ethnopharmacol..

[199] Choi G.Y., Kim H.B., Hwang E.S., Lee S., Kim M.J., Choi J.Y., Lee S.O., Kim S.S., Park J.H. (2017). Curcumin Alters Neural Plasticity and Viability of Intact Hippocampal Circuits and Attenuates Behavioral Despair and COX-2 Expression in Chronically Stressed Rats. Mediat. Inflamm..

[200] Mao Q.Q., Xian Y.F., Ip S.P., Che C.T. (2011). Involvement of serotonergic system in the antidepressant-like effect of piperine. Prog. Neuro-Psychopharmacol. Biol. Psychiatry.

[201] Mao Q.Q., Huang Z., Zhong X.M., Xian Y.F., Ip S.P. (2014). Brain-derived neurotrophic factor signalling mediates the antidepressant-like effect of piperine in chronically stressed mice. Behav. Brain Res..

[202] Lin S., Li Q., Jiang S., Xu Z., Jiang Y., Liu L., Jiang J., Tong Y., Wang P. (2021). Crocetin ameliorates chronic restraint stress-induced depression-like behaviors in mice by regulating MEK/ERK pathways and gut microbiota. J. Ethnopharmacol..

[203] Yan T., Xu M., Wu B., Liao Z., Liu Z., Zhao X., Bi K., Jia Y. (2016). The effect of Schisandra chinensis extracts on depression by noradrenergic, dopaminergic, GABAergic and glutamatergic systems in the forced swim test in mice. Food Funct..

[204] Chen S., Tang Y., Gao Y., Nie K., Wang H., Su H., Wang Z., Lu F., Huang W., Dong H. (2022). Antidepressant Potential of Quercetin and its Glycoside Derivatives: A Comprehensive Review and Update. Front. Pharmacol..

[205] Calder P.C. (1998). Immunoregulatory and anti-inflammatory effects of n-3 polyunsaturated fatty acids. Braz. J. Med. Biol. Res..

[206] Miralles-Perez B., Mendez L., Nogues M.R., Sanchez-Martos V., Fortuno-Mar A., Ramos-Romero S., Hereu M., Medina I., Romeu M. (2021). Effects of a Fish Oil Rich in Docosahexaenoic Acid on Cardiometabolic Risk Factors and Oxidative Stress in Healthy Rats. Mar. Drugs.

[207] Vines A., Delattre A.M., Lima M.M., Rodrigues L.S., Suchecki D., Machado R.B., Tufik S., Pereira S.I., Zanata S.M., Ferraz A.C. (2012). The role of 5-HT_1A_ receptors in fish oil-mediated increased BDNF expression in the rat hippocampus and cortex: A possible antidepressant mechanism. Neuropharmacology.

[208] Pardo Lozano R., Alvarez Garcia Y., Barral Tafalla D., Farre Albaladejo M. (2007). Caffeine: A nutrient, a drug or a drug of abuse. Adicciones.

[209] Faudone G., Arifi S., Merk D. (2021). The Medicinal Chemistry of Caffeine. J. Med. Chem..

[210] Lopez-Cruz L., Salamone J.D., Correa M. (2018). Caffeine and Selective Adenosine Receptor Antagonists as New Therapeutic Tools for the Motivational Symptoms of Depression. Front. Pharmacol..

[211] Boros L.G. (2000). Population thiamine status and varying cancer rates between western, Asian and African countries. Anticancer Res..

[212] Sambon M., Wins P., Bettendorff L. (2021). Neuroprotective Effects of Thiamine and Precursors with Higher Bioavailability: Focus on Benfotiamine and Dibenzoylthiamine. Int. J. Mol. Sci..

[213] Mosegaard S., Dipace G., Bross P., Carlsen J., Gregersen N., Olsen R.K.J. (2020). Riboflavin Deficiency-Implications for General Human Health and Inborn Errors of Metabolism. Int. J. Mol. Sci..

[214] Huang S.K., Lu C.W., Lin T.Y., Wang S.J. (2022). Neuroprotective Role of the B Vitamins in the Modulation of the Central Glutamatergic Neurotransmission. CNS Neurol. Disord. Drug Targets.

[215] Dosedel M., Jirkovsky E., Macakova K., Krcmova L.K., Javorska L., Pourova J., Mercolini L., Remiao F., Novakova L., Mladenka P. (2021). Vitamin C-Sources, Physiological Role, Kinetics, Deficiency, Use, Toxicity, and Determination. Nutrients.

[216] Moritz B., Schmitz A.E., Rodrigues A.L.S., Dafre A.L., Cunha M.P. (2020). The role of vitamin C in stress-related disorders. J. Nutr. Biochem..

[217] Benedik E. (2022). Sources of vitamin D for humans. Int. J. Vitam. Nutr. Res..

[218] Urena-Torres P., Souberbielle J.C. (2014). Pharmacologic role of vitamin D natural products. Curr. Vasc. Pharmacol..

[219] Kouba B.R., Camargo A., Gil-Mohapel J., Rodrigues A.L.S. (2022). Molecular Basis Underlying the Therapeutic Potential of Vitamin D for the Treatment of Depression and Anxiety. Int. J. Mol. Sci..

[220] Ni X.C., Wang H.F., Cai Y.Y., Yang D., Alolga R.N., Liu B., Li J., Huang F.Q. (2022). Ginsenoside Rb1 inhibits astrocyte activation and promotes transfer of astrocytic mitochondria to neurons against ischemic stroke. Redox Biol..

[221] Gong L., Yin J., Zhang Y., Huang R., Lou Y., Jiang H., Sun L., Jia J., Zeng X. (2022). Neuroprotective Mechanisms of Ginsenoside Rb1 in Central Nervous System Diseases. Front. Pharmacol..

[222] Guo Y., Xie J., Zhang L., Yang L., Ma J., Bai Y., Ma W., Wang L., Yu H., Zeng Y. (2021). Ginsenoside Rb1 exerts antidepressant-like effects via suppression inflammation and activation of AKT pathway. Neurosci. Lett..

[223] Matias J.N., Achete G., Campanari G., Guiguer E.L., Araujo A.C., Buglio D.S., Barbalho S.M. (2021). A systematic review of the antidepressant effects of curcumin: Beyond monoamines theory. Aust. N. Z. J. Psychiatry.

[224] Marton L.T., Pescinini E.S.L.M., Camargo M.E.C., Barbalho S.M., Haber J., Sinatora R.V., Detregiachi C.R.P., Girio R.J.S., Buchaim D.V., Cincotto Dos Santos Bueno P. (2021). The Effects of Curcumin on Diabetes Mellitus: A Systematic Review. Front. Endocrinol..

[225] Imran M., Samal M., Qadir A., Ali A., Mir S.R. (2022). A critical review on the extraction and pharmacotherapeutic activity of piperine. Polim. Med..

[226] Li S., Wang C., Wang M., Li W., Matsumoto K., Tang Y. (2007). Antidepressant like effects of piperine in chronic mild stress treated mice and its possible mechanisms. Life Sci..

[227] Glabska D., Guzek D., Groele B., Gutkowska K. (2020). Fruit and Vegetable Intake and Mental Health in Adults: A Systematic Review. Nutrients.

[228] McCarty M.F. (2008). Scavenging of peroxynitrite-derived radicals by flavonoids may support endothelial NO synthase activity, contributing to the vascular protection associated with high fruit and vegetable intakes. Med. Hypotheses.

[229] Bishwajit G., O’Leary D.P., Ghosh S., Sanni Y., Shangfeng T., Zhanchun F. (2017). Association between depression and fruit and vegetable consumption among adults in South Asia. BMC Psychiatry.

[230] Richard A., Rohrmann S., Vandeleur C.L., Mohler-Kuo M., Eichholzer M. (2015). Associations between fruit and vegetable consumption and psychological distress: Results from a population-based study. BMC Psychiatry.

[231] Song H., Shen X., Chu Q., Zheng X. (2021). Vaccinium bracteatum Thunb. fruit extract reduces high-fat diet-induced obesity with modulation of the gut microbiota in obese mice. J. Food Biochem..

[232] Reddy A.J., Handu S.S., Dubey A.K., Mediratta P.K., Shukla R., Ahmed Q.M. (2016). Effect of Musa sapientum Stem Extract on Animal Models of Depression. Pharmacogn. Res..

[233] Hu H., Wang J., Hu Y., Xie J. (2020). Nutritional component changes in Xiangfen 1 banana at different developmental stages. Food Funct..

[234] Valdes-Sustaita B., Estrada-Camarena E., Gonzalez-Trujano M.E., Lopez-Rubalcava C. (2021). Estrogen receptors-beta and serotonin mediate the antidepressant-like effect of an aqueous extract of pomegranate in ovariectomized rats. Neurochem. Int..

[235] Mori-Okamoto J., Otawara-Hamamoto Y., Yamato H., Yoshimura H. (2004). Pomegranate extract improves a depressive state and bone properties in menopausal syndrome model ovariectomized mice. J. Ethnopharmacol..

[236] Li Y., Yao J., Han C., Yang J., Chaudhry M.T., Wang S., Liu H., Yin Y. (2016). Quercetin, Inflammation and Immunity. Nutrients.

[237] Han X.J., Xu T.S., Fang Q.J., Zhang H.J., Yue L.J., Hu G., Sun L.Y. (2021). Quercetin hinders microglial activation to alleviate neurotoxicity via the interplay between NLRP3 inflammasome and mitophagy. Redox Biol..

[238] Kohno M., Hirotsuka M., Kito M., Matsuzawa Y. (2006). Decreases in serum triacylglycerol and visceral fat mediated by dietary soybean beta-conglycinin. J. Atheroscler. Thromb..

[239] Wu S.J., Chang C.Y., Lai Y.T., Shyu Y.T. (2020). Increasing gamma-Aminobutyric Acid Content in Vegetable Soybeans via High-Pressure Processing and Efficacy of Their Antidepressant-Like Activity in Mice. Foods.

[240] Ji W.W., Li R.P., Li M., Wang S.Y., Zhang X., Niu X.X., Li W., Yan L., Wang Y., Fu Q. (2014). Antidepressant-like effect of essential oil of Perilla frutescens in a chronic, unpredictable, mild stress-induced depression model mice. Chin. J. Nat. Med..

[241] Wang A., Wan X., Zhuang P., Jia W., Ao Y., Liu X., Tian Y., Zhu L., Huang Y., Yao J. (2023). High fried food consumption impacts anxiety and depression due to lipid metabolism disturbance and neuroinflammation. Proc. Natl. Acad. Sci. USA.

[242] Philippou E., Nikiphorou E. (2018). Are we really what we eat? Nutrition and its role in the onset of rheumatoid arthritis. Autoimmun. Rev..

[243] Sharifan P., Hosseini M.S., Sharifan A. (2017). The interventional relationship between frequent fish consumption and depression symptoms in aging adults: A randomized controlled trial. Int. J. Geriatr. Psychiatry.

[244] Yang C.P., Chang C.M., Yang C.C., Pariante C.M., Su K.P. (2022). Long COVID and long chain fatty acids (LCFAs): Psychoneuroimmunity implication of omega-3 LCFAs in delayed consequences of COVID-19. Brain Behav. Immun..

[245] Wu A., Ying Z., Gomez-Pinilla F. (2004). Dietary omega-3 fatty acids normalize BDNF levels, reduce oxidative damage, and counteract learning disability after traumatic brain injury in rats. J. Neurotrauma.

[246] Nasehi M., Mosavi-Nezhad S.M., Khakpai F., Zarrindast M.R. (2018). The role of omega-3 on modulation of cognitive deficiency induced by REM sleep deprivation in rats. Behav. Brain Res..

[247] Simopoulos A.P. (2016). An Increase in the Omega-6/Omega-3 Fatty Acid Ratio Increases the Risk for Obesity. Nutrients.

[248] Whiting C.V., Bland P.W., Tarlton J.F. (2005). Dietary n-3 polyunsaturated fatty acids reduce disease and colonic proinflammatory cytokines in a mouse model of colitis. Inflamm. Bowel Dis..

[249] Parletta N., Zarnowiecki D., Cho J., Wilson A., Bogomolova S., Villani A., Itsiopoulos C., Niyonsenga T., Blunden S., Meyer B. (2019). A Mediterranean-style dietary intervention supplemented with fish oil improves diet quality and mental health in people with depression: A randomized controlled trial (HELFIMED). Nutr. Neurosci..

[250] Naliwaiko K., Araujo R.L., da Fonseca R.V., Castilho J.C., Andreatini R., Bellissimo M.I., Oliveira B.H., Martins E.F., Curi R., Fernandes L.C. (2004). Effects of fish oil on the central nervous system: A new potential antidepressant?. Nutr. Neurosci..

[251] Davis D.J., Hecht P.M., Jasarevic E., Beversdorf D.Q., Will M.J., Fritsche K., Gillespie C.H. (2017). Sex-specific effects of docosahexaenoic acid (DHA) on the microbiome and behavior of socially-isolated mice. Brain Behav. Immun..

[252] Pusceddu M.M., El Aidy S., Crispie F., O’Sullivan O., Cotter P., Stanton C., Kelly P., Cryan J.F., Dinan T.G. (2015). N-3 Polyunsaturated Fatty Acids (PUFAs) Reverse the Impact of Early-Life Stress on the Gut Microbiota. PLoS ONE.

[253] Grosso G., Galvano F., Marventano S., Malaguarnera M., Bucolo C., Drago F., Caraci F. (2014). Omega-3 fatty acids and depression: Scientific evidence and biological mechanisms. Oxid. Med. Cell Longev..

[254] Caroprese M., Ciliberti M.G., Annicchiarico G., Albenzio M., Muscio A., Sevi A. (2014). Hypothalamic-pituitary-adrenal axis activation and immune regulation in heat-stressed sheep after supplementation with polyunsaturated fatty acids. J. Dairy Sci..

[255] Feng J., Wang Q., Yang W., Liu J., Gao M.Q. (2021). Omega-3 polyunsaturated fatty acids ameliorated inflammatory response of mammary epithelial cells and mammary gland induced by lipopolysaccharide. Acta Biochim. Biophys. Sin..

[256] Takeuchi E., Yamada D., Suzuki S., Saitoh A., Itoh M., Hayashi T., Yamada M., Wada K., Sekiguchi M. (2020). Participation of the nucleus accumbens dopaminergic system in the antidepressant-like actions of a diet rich in omega-3 polyunsaturated fatty acids. PLoS ONE.

[257] Naeem S., Ali L., Rizwani G.H., Ikram R., Khan S.S., Shareef H., Younus I., Malick T.Z., Aleem U. (2020). A comparative neurobehavioral study of sesame oil and fish oil on experimental animals. Pak. J. Pharm. Sci..

[258] Ferraz A.C., Kiss A., Araujo R.L., Salles H.M., Naliwaiko K., Pamplona J., Matheussi F. (2008). The antidepressant role of dietary long-chain polyunsaturated n-3 fatty acids in two phases in the developing brain. Prostaglandins Leukot. Essent. Fat. Acids.

[259] Wu B., Song Q., Zhang Y., Wang C., Yang M., Zhang J., Han W., Jiang P. (2020). Antidepressant activity of omega-3 polyunsaturated fatty acids in ovariectomized rats: Role of neuroinflammation and microglial polarization. Lipids Health Dis..

[260] Correa C.R., Schena C., Lopes S.C., Prediger R.D., Silva E.L., Venske D.K.R., Ribeiro L.C., Moreira J.D. (2020). Combined effects of caloric restriction and fish oil attenuated anti-depressant and anxiolytic-like effects of fish oil: Association with hippocampal BDNF concentrations. Behav. Brain Res..

[261] Carabelli B., Delattre A.M., Pudell C., Mori M.A., Suchecki D., Machado R.B., Venancio D.P., Piazzetta S.R., Hammerschmidt I., Zanata S.M. (2015). The Antidepressant-Like Effect of Fish Oil: Possible Role of Ventral Hippocampal 5-HT_1A_ Post-synaptic Receptor. Mol. Neurobiol..

[262] Costantini L., Molinari R., Farinon B., Merendino N. (2017). Impact of Omega-3 Fatty Acids on the Gut Microbiota. Int. J. Mol. Sci..

[263] Appleton K.M., Voyias P.D., Sallis H.M., Dawson S., Ness A.R., Churchill R., Perry R. (2021). Omega-3 fatty acids for depression in adults. Cochrane Database Syst. Rev..

[264] Qi Y., Zhang H., Liang S., Chen J., Yan X., Duan Z., Zhou D., Li Z. (2020). Evaluation of the Antidepressant Effect of the Functional Beverage Containing Active Peptides, Menthol and Eleutheroside and Investigation of Its Mechanism of Action in Mice. Food Technol. Biotechnol..

[265] Yin Y.Q., Zhang C., Wang J.X., Hou J., Yang X., Qin J. (2015). Chronic caffeine treatment enhances the resilience to social defeat stress in mice. Food Funct..

[266] Szopa A., Doboszewska U., Herbet M., Wosko S., Wyska E., Swiader K., Serefko A., Korga A., Wlaz A., Wrobel A. (2017). Chronic treatment with caffeine and its withdrawal modify the antidepressant-like activity of selective serotonin reuptake inhibitors in the forced swim and tail suspension tests in mice. Effects on Comt, Slc6a15 and Adora1 gene expression. Toxicol. Appl. Pharmacol..

[267] Kale P.P., Addepalli V. (2014). Augmentation of antidepressant effects of duloxetine and bupropion by caffeine in mice. Pharmacol. Biochem. Behav..

[268] Liu Q.S., Deng R., Fan Y., Li K., Meng F., Li X., Liu R. (2017). Low dose of caffeine enhances the efficacy of antidepressants in major depressive disorder and the underlying neural substrates. Mol. Nutr. Food Res..

[269] Fredholm B.B., Battig K., Holmen J., Nehlig A., Zvartau E.E. (1999). Actions of caffeine in the brain with special reference to factors that contribute to its widespread use. Pharmacol. Rev..

[270] Serefko A., Szopa A., Wlaz A., Wosko S., Wlaz P., Poleszak E. (2016). Synergistic antidepressant-like effect of the joint administration of caffeine and NMDA receptor ligands in the forced swim test in mice. J. Neural Transm..

[271] Espinosa Jovel C.A., Sobrino Mejia F.E. (2017). Caffeine and headache: Specific remarks. Neurologia.

[272] Meller F.O., Manosso L.M., Schafer A.A. (2021). The influence of diet quality on depression among adults and elderly: A population-based study. J. Affect. Disord..

[273] Rothenberg D.O., Zhang L. (2019). Mechanisms Underlying the Anti-Depressive Effects of Regular Tea Consumption. Nutrients.

[274] Shao J., Wei Y., Wei X. (2022). A comprehensive review on bioavailability, safety and antidepressant potential of natural bioactive components from tea. Food Res. Int..

[275] Voskoboinik A., Koh Y., Kistler P.M. (2019). Cardiovascular effects of caffeinated beverages. Trends Cardiovasc. Med..

[276] Teng J., Zhou W., Zeng Z., Zhao W., Huang Y., Zhang X. (2017). Quality components and antidepressant-like effects of GABA green tea. Food Funct..

[277] Zhu W.L., Shi H.S., Wei Y.M., Wang S.J., Sun C.Y., Ding Z.B., Lu L. (2012). Green tea polyphenols produce antidepressant-like effects in adult mice. Pharmacol. Res..

[278] Linnoila M.I. (1989). Anxiety and alcoholism. J. Clin. Psychiatry.

[279] Ciccocioppo R., Panocka I., Froldi R., Colombo G., Gessa G.L., Massi M. (1999). Antidepressant-like effect of ethanol revealed in the forced swimming test in Sardinian alcohol-preferring rats. Psychopharmacology.

[280] Gea A., Beunza J.J., Estruch R., Sanchez-Villegas A., Salas-Salvado J., Buil-Cosiales P., Gomez-Gracia E., Covas M.I., Corella D., Fiol M. (2013). Alcohol intake, wine consumption and the development of depression: The PREDIMED study. BMC Med..

[281] Bonnet U. (2017). How much alcohol is in ketamine’s antidepressant action?. Life Sci..

[282] Leclercq S., Le Roy T., Furgiuele S., Coste V., Bindels L.B., Leyrolle Q., Neyrinck A.M., Quoilin C., Amadieu C., Petit G. (2020). Gut Microbiota-Induced Changes in beta-Hydroxybutyrate Metabolism Are Linked to Altered Sociability and Depression in Alcohol Use Disorder. Cell Rep..

[283] Morkl S., Butler M.I., Holl A., Cryan J.F., Dinan T.G. (2020). Probiotics and the Microbiota-Gut-Brain Axis: Focus on Psychiatry. Curr. Nutr. Rep..

[284] Silva L.C., de Souza Lago H., Rocha M.O.T., de Oliveira V.S., Laureano-Melo R., Stutz E.T.G., de Paula B.P., Martins J.F.P., Luchese R.H., Guerra A.F. (2021). Craft Beers Fermented by Potential Probiotic Yeast or Lacticaseibacilli Strains Promote Antidepressant-Like Behavior in Swiss Webster Mice. Probiotics Antimicrob. Proteins.

[285] Chen H.-L., Lan Y.-W., Tu M.-Y., Tung Y.-T., Chan M.N.-Y., Wu H.-S., Yen C.-C., Chen C.-M. (2021). Kefir peptides exhibit antidepressant-like activity in mice through the BDNF/TrkB pathway. J. Dairy Sci..

[286] Kim C.S., Cha L., Sim M., Jung S., Chun W.Y., Baik H.W., Shin D.M. (2021). Probiotic Supplementation Improves Cognitive Function and Mood with Changes in Gut Microbiota in Community-Dwelling Older Adults: A Randomized, Double-Blind, Placebo-Controlled, Multicenter Trial. J. Gerontol. A Biol. Sci. Med. Sci..

[287] Abildgaard A., Solskov L., Volke V., Harvey B.H., Lund S., Wegener G. (2011). A high-fat diet exacerbates depressive-like behavior in the Flinders Sensitive Line (FSL) rat, a genetic model of depression. Psychoneuroendocrinology.

[288] Abildgaard A., Kern T., Pedersen O., Hansen T., Lund S., Wegener G. (2021). A diet-induced gut microbiota component and related plasma metabolites are associated with depressive-like behaviour in rats. Eur. Neuropsychopharmacol..

[289] Tillmann S., Awwad H.M., Eskelund A.R., Treccani G., Geisel J., Wegener G., Obeid R. (2018). Probiotics Affect One-Carbon Metabolites and Catecholamines in a Genetic Rat Model of Depression. Mol. Nutr. Food Res..

[290] Cheng L.H., Liu Y.W., Wu C.C., Wang S., Tsai Y.C. (2019). Psychobiotics in mental health, neurodegenerative and neurodevelopmental disorders. J. Food Drug Anal..

[291] Mikkelsen K., Stojanovska L., Apostolopoulos V. (2016). The Effects of Vitamin B in Depression. Curr. Med. Chem..

[292] Green R., Miller J.W. (2022). Vitamin B12 deficiency. Vitam. Horm..

[293] Wang X., Wang T., Sun L., Zhang H., Liu C., Zhang C., Yu L. (2020). B-vitamin supplementation ameliorates anxiety- and depression-like behavior induced by gestational urban PM_2.5_ exposure through suppressing neuroinflammation in mice offspring. Environ. Pollut..

[294] Mesripour A., Alhimma F., Hajhashemi V. (2019). The effect of vitamin B6 on dexamethasone-induced depression in mice model of despair. Nutr. Neurosci..

[295] Watanabe F., Yabuta Y., Bito T., Teng F. (2014). Vitamin B_12_-containing plant food sources for vegetarians. Nutrients.

[296] Coppen A., Bolander-Gouaille C. (2005). Treatment of depression: Time to consider folic acid and vitamin B12. J. Psychopharmacol..

[297] Brocardo P.S., Budni J., Kaster M.P., Santos A.R., Rodrigues A.L. (2008). Folic acid administration produces an antidepressant-like effect in mice: Evidence for the involvement of the serotonergic and noradrenergic systems. Neuropharmacology.

[298] Rosa P.B., Ribeiro C.M., Bettio L.E., Colla A., Lieberknecht V., Moretti M., Rodrigues A.L. (2014). Folic acid prevents depressive-like behavior induced by chronic corticosterone treatment in mice. Pharmacol. Biochem. Behav..

[299] Thomas J., Khanam R., Vohora D. (2016). Augmentation of effect of venlafaxine by folic acid in behavioral paradigms of depression in mice: Evidence of serotonergic and pro-inflammatory cytokine pathways. Pharmacol. Rep..

[300] Carr A.C., Maggini S. (2017). Vitamin C and Immune Function. Nutrients.

[301] Riaz A., Khan R.A. (2017). Behavioral effects of Citrus limon and Punica granatum combinations in rats. Metab. Brain Dis..

[302] Binfare R.W., Rosa A.O., Lobato K.R., Santos A.R., Rodrigues A.L. (2009). Ascorbic acid administration produces an antidepressant-like effect: Evidence for the involvement of monoaminergic neurotransmission. Prog. Neuro-Psychopharmacol. Biol. Psychiatry.

[303] Rosa P.B., Neis V.B., Ribeiro C.M., Moretti M., Rodrigues A.L. (2016). Antidepressant-like effects of ascorbic acid and ketamine involve modulation of GABAA and GABAB receptors. Pharmacol. Rep..

[304] Fraga D.B., Olescowicz G., Moretti M., Siteneski A., Tavares M.K., Azevedo D., Colla A.R.S., Rodrigues A.L.S. (2018). Anxiolytic effects of ascorbic acid and ketamine in mice. J. Psychiatr. Res..

[305] Meredith M.E., May J.M. (2013). Regulation of embryonic neurotransmitter and tyrosine hydroxylase protein levels by ascorbic acid. Brain Res..

[306] Zech L.D., Scherf-Clavel M., Daniels C., Schwab M., Deckert J., Unterecker S., Herr A.S. (2021). Patients with higher vitamin D levels show stronger improvement of self-reported depressive symptoms in psychogeriatric day-care setting. J. Neural Transm..

[307] Wyskida M., Wieczorowska-Tobis K., Chudek J. (2017). Prevalence and factors promoting the occurrence of vitamin D deficiency in the elderly. Postep. Hig. I Med. Dosw. Online.

[308] Glade M.J. (2013). Vitamin D: Health panacea or false prophet?. Nutrition.

[309] Chuang H.W., Wei I.H., Lin F.Y., Li C.T., Chen K.T., Tsai M.H., Huang C.C. (2020). Roles of Akt and ERK in mTOR-Dependent Antidepressant Effects of Vanillic Acid. ACS Omega.

[310] Moradi O. (2022). A review on nanomaterial-based electrochemical sensors for determination of vanillin in food samples. Food Chem. Toxicol..

[311] Yang W., Qiu X., Wu Q., Chang F., Zhou T., Zhou M., Pei J. (2023). Active constituents of saffron (*Crocus sativus* L.) and their prospects in treating neurodegenerative diseases (Review). Exp. Ther. Med..

[312] Sahib N.G., Anwar F., Gilani A.H., Hamid A.A., Saari N., Alkharfy K.M. (2013). Coriander (*Coriandrum sativum* L.): A potential source of high-value components for functional foods and nutraceuticals—A review. Phytother. Res..

[313] Wang Y.S., Shen C.Y., Jiang J.G. (2019). Antidepressant active ingredients from herbs and nutraceuticals used in TCM: Pharmacological mechanisms and prospects for drug discovery. Pharmacol. Res..

[314] Jin Y., Cui R., Zhao L., Fan J., Li B. (2019). Mechanisms of Panax ginseng action as an antidepressant. Cell Prolif..

[315] Jiang N., Wang H., Li C., Zeng G., Lv J., Wang Q., Chen Y., Liu X. (2021). The antidepressant-like effects of the water extract of Panax ginseng and Polygala tenuifolia are mediated via the BDNF-TrkB signaling pathway and neurogenesis in the hippocampus. J. Ethnopharmacol..

[316] Yamada N., Araki H., Yoshimura H. (2011). Identification of antidepressant-like ingredients in ginseng root (*Panax ginseng* C.A. Meyer) using a menopausal depressive-like state in female mice: Participation of 5-HT_2A_ receptors. Psychopharmacology.

[317] Sanmukhani J., Anovadiya A., Tripathi C.B. (2011). Evaluation of antidepressant like activity of curcumin and its combination with fluoxetine and imipramine: An acute and chronic study. Acta Pol. Pharm..

[318] Abd-Rabo M.M., Georgy G.S., Saied N.M., Hassan W.A. (2019). Involvement of the serotonergic system and neuroplasticity in the antidepressant effect of curcumin in ovariectomized rats: Comparison with oestradiol and fluoxetine. Phytother. Res..

[319] Lian L., Xu Y., Zhang J., Yu Y., Zhu N., Guan X., Huang H., Chen R., Chen J., Shi G. (2018). Antidepressant-like effects of a novel curcumin derivative J147: Involvement of 5-HT1A receptor. Neuropharmacology.

[320] Quijia C.R., Chorilli M. (2020). Characteristics, Biological Properties and Analytical Methods of Piperine: A Review. Crit. Rev. Anal. Chem..

[321] Rybnikar M., Smejkal K., Zemlicka M. (2019). Schisandra chinensis and its phytotherapeutical applications. Ceska A Slov. Farm..

[322] Yan T., He B., Wan S., Xu M., Yang H., Xiao F., Bi K., Jia Y. (2017). Antidepressant-like effects and cognitive enhancement of Schisandra chinensis in chronic unpredictable mild stress mice and its related mechanism. Sci. Rep..

[323] Song Y., Shan B., Zeng S., Zhang J., Jin C., Liao Z., Wang T., Zeng Q., He H., Wei F. (2021). Raw and wine processed Schisandra chinensis attenuate anxiety like behavior via modulating gut microbiota and lipid metabolism pathway. J. Ethnopharmacol..

[324] Tomonaga S., Yamane H., Onitsuka E., Yamada S., Sato M., Takahata Y., Morimatsu F., Furuse M. (2008). Carnosine-induced antidepressant-like activity in rats. Pharmacol. Biochem. Behav..

[325] Jacka F.N., Mykletun A., Berk M., Bjelland I., Tell G.S. (2011). The association between habitual diet quality and the common mental disorders in community-dwelling adults: The Hordaland Health study. Psychosom. Med..

[326] Nagasawa M., Murakami T., Sato M., Takahata Y., Morimatsu F., Furuse M. (2012). Dietary animal proteins alter monoamine metabolism in the brain. Anim. Sci. J..

[327] Huang F., Zhang J., Jia X., Huang X., Wu Z., Shi X., Zhao J., Liu S., Ding G. (2021). Association between dietary patterns and depressive symptom based on reduced rank regression in people aged 55 and above in 4 provinces of China. Wei Sheng Yan Jiu.

[328] Shimoyoshi S., Takemoto D., Ono Y., Kitagawa Y., Shibata H., Tomono S., Unno K., Wakabayashi K. (2019). Sesame Lignans Suppress Age-Related Cognitive Decline in Senescence-Accelerated Mice. Nutrients.

[329] Wang Q., Jia M., Zhao Y., Hui Y., Pan J., Yu H., Yan S., Dai X., Liu X., Liu Z. (2019). Supplementation of Sesamin Alleviates Stress-Induced Behavioral and Psychological Disorders via Reshaping the Gut Microbiota Structure. J. Agric. Food Chem..

[330] Zhao Y., Wang Q., Jia M., Fu S., Pan J., Chu C., Liu X., Liu X., Liu Z. (2019). (+)-Sesamin attenuates chronic unpredictable mild stress-induced depressive-like behaviors and memory deficits via suppression of neuroinflammation. J. Nutr. Biochem..

[331] Lee H.C., Ko H.K., Huang B.E., Chu Y.H., Huang S.Y. (2014). Antidepressant-like effects of Perilla frutescens seed oil during a forced swimming test. Food Funct..

[332] Lu X., Ce Q., Jin L., Zheng J., Sun M., Tang X., Li D., Sun J. (2021). Deoiled sunflower seeds ameliorate depression by promoting the production of monoamine neurotransmitters and inhibiting oxidative stress. Food Funct..

[333] Wu Z., Wang P., Pan D., Zeng X., Guo Y., Zhao G. (2021). Effect of adzuki bean sprout fermented milk enriched in gamma-aminobutyric acid on mild depression in a mouse model. J. Dairy Sci..

[334] Ano Y., Ohya R., Kondo K. (2019). Antidepressant-Like Effect of beta-Lactolin, a Glycine-Threonine-Tryptophan-Tyrosine Peptide. J. Nutr. Sci. Vitaminol..

[335] Yun B., Yoo J.Y., Park M.R., Ryu S., Lee W.J., Choi H.J., Kang M.K., Kim Y., Oh S. (2020). Ingestion of Gouda Cheese Ameliorates the Chronic Unpredictable Mild Stress in Mice. Food Sci. Anim. Resour..

[336] Cui Y., Huang C., Momma H., Ren Z., Sugiyama S., Guan L., Niu K., Nagatomi R. (2017). Consumption of low-fat dairy, but not whole-fat dairy, is inversely associated with depressive symptoms in Japanese adults. Soc. Psychiatry Psychiatr. Epidemiol..

[337] Sun J., Wang W., Zhang D. (2020). Associations of different types of dairy intakes with depressive symptoms in adults. J. Affect. Disord..

[338] Mahdavifar B., Hosseinzadeh M., Salehi-Abargouei A., Mirzaei M., Vafa M. (2022). The association between dairy products and psychological disorders in a large sample of Iranian adults. Nutr. Neurosci..

[339] Qian J., Fang Y., Yuan N., Gao X., Lv Y., Zhao C., Zhang S., Li Q., Li L., Xu L. (2021). Innate immune remodeling by short-term intensive fasting. Aging Cell.

[340] Spanaki C., Rodopaios N.E., Koulouri A., Pliakas T., Papadopoulou S.K., Vasara E., Skepastianos P., Serafeim T., Boura I., Dermitzakis E. (2021). The Christian Orthodox Church Fasting Diet Is Associated with Lower Levels of Depression and Anxiety and a Better Cognitive Performance in Middle Life. Nutrients.

[341] Gomes A.P., Oliveira Bierhals I., Goncalves Soares A.L., Hellwig N., Tomasi E., Formoso Assuncao M.C., Goncalves H. (2018). Interrelatioship between Diet Quality and Depressive Symptoms in Elderly. J. Nutr. Health Aging.

[342] Rudzinska A., Perera I., Gryglewska B., Gasowski J., Piotrowicz K. (2022). Can the Mediterranean diet decrease the risk of depression in older persons—A systematic review. Psychiatr. Pol..

[343] Li Y., Lv M.R., Wei Y.J., Sun L., Zhang J.X., Zhang H.G., Li B. (2017). Dietary patterns and depression risk: A meta-analysis. Psychiatry Res..

[344] Santos C.J., Ferreira A.V.M., Oliveira A.L., Oliveira M.C., Gomes J.S., Aguiar D.C. (2018). Carbohydrate-enriched diet predispose to anxiety and depression-like behavior after stress in mice. Nutr. Neurosci..

[345] Mezuk B., Eaton W.W., Albrecht S., Golden S.H. (2008). Depression and type 2 diabetes over the lifespan: A meta-analysis. Diabetes Care.

[346] Linehan V., Fang L.Z., Hirasawa M. (2018). Short-term high-fat diet primes excitatory synapses for long-term depression in orexin neurons. J. Physiol..

[347] Dutheil S., Ota K.T., Wohleb E.S., Rasmussen K., Duman R.S. (2015). High-Fat Diet Induced Anxiety and Anhedonia: Impact on Brain Homeostasis and Inflammation. Neuropsychopharmacology.

[348] Dakanalis A., Mentzelou M., Papadopoulou S.K., Papandreou D., Spanoudaki M., Vasios G.K., Pavlidou E., Mantzorou M., Giaginis C. (2023). The Association of Emotional Eating with Overweight/Obesity, Depression, Anxiety/Stress, and Dietary Patterns: A Review of the Current Clinical Evidence. Nutrients.

[349] Schachter J., Martel J., Lin C.S., Chang C.J., Wu T.R., Lu C.C., Ko Y.F., Lai H.C., Ojcius D.M., Young J.D. (2018). Effects of obesity on depression: A role for inflammation and the gut microbiota. Brain Behav. Immun..

[350] Martins L.B., Monteze N.M., Calarge C., Ferreira A.V.M., Teixeira A.L. (2019). Pathways linking obesity to neuropsychiatric disorders. Nutrition.

[351] Ali A.T., Hochfeld W.E., Myburgh R., Pepper M.S. (2013). Adipocyte and adipogenesis. Eur. J. Cell Biol..

[352] Weiss G.A., Hennet T. (2017). Mechanisms and consequences of intestinal dysbiosis. Cell. Mol. Life Sci. CMLS.

[353] Jayaraj R.L., Azimullah S., Beiram R., Jalal F.Y., Rosenberg G.A. (2019). Neuroinflammation: Friend and foe for ischemic stroke. J. Neuroinflamm..

[354] Nedic Erjavec G., Sagud M., Nikolac Perkovic M., Svob Strac D., Konjevod M., Tudor L., Uzun S., Pivac N. (2021). Depression: Biological markers and treatment. Prog. Neuro-Psychopharmacol. Biol. Psychiatry.

[355] Milaneschi Y., Simmons W.K., van Rossum E.F.C., Penninx B.W. (2019). Depression and obesity: Evidence of shared biological mechanisms. Mol. Psychiatry.

[356] Li B., Zhao J., Lv J., Tang F., Liu L., Sun Z., Wang L., Siwela S.P., Wang Y., Song Y. (2014). Additive antidepressant-like effects of fasting with imipramine via modulation of 5-HT_2_ receptors in the mice. Prog. Neuro-Psychopharmacol. Biol. Psychiatry.

[357] Wang P., Li B., Fan J., Zhang K., Yang W., Ren B., Cui R. (2019). Additive antidepressant-like effects of fasting with beta-estradiol in mice. J. Cell Mol. Med..

[358] Bear T.L.K., Dalziel J.E., Coad J., Roy N.C., Butts C.A., Gopal P.K. (2020). The Role of the Gut Microbiota in Dietary Interventions for Depression and Anxiety. Adv. Nutr..

